# Isolation of Potassium Bis(amido)diazadipnictogenide Salts

**DOI:** 10.1021/acs.organomet.5c00493

**Published:** 2026-01-26

**Authors:** Reece Lister-Roberts, Meera Mehta

**Affiliations:** † Department of Chemistry, 6396 University of Oxford, 12 Mansfield Road, Oxford OX1 3TA, U.K.; ‡ Department of Chemistry, University of Manchester, Oxford Road, Manchester M13 9PL, U.K.

## Abstract

The reaction of Zintl phases K_3_As_7_ and K_3_P_7_ were investigated with 1-azido-4-bromobenzene (4-BrC_6_H_4_N_3_) and found to yield potassium salts of substituted diazadipnictogen rings (general molecular fragment {(RN)_2_Pn_2_}; Pn = P, As). In the case of the arsenic derivatives, amido­(imino)­arsenide persists in solution and bis­(amido)­diazadiarsenide persists in the solid state. These two species can be considered to share a monomer–dimer relationship, accessible by alteration between the solid and solution states, whereas in the case of K_3_P_7_, our crystallographic and spectroscopic studies are consistent with the formation of a bisamido­(bisimino)­diazadiphosphenide, the “dimeric” form, persisting in both the solid and solution phase. Subsequent reactivity studies with amido­(imino)­arsenide show that it can act as a source of amide anion, giving a carbonate and a thiourea derivative when investigated with CO_2_ and CS_2_, respectively.

## Graphical abstract

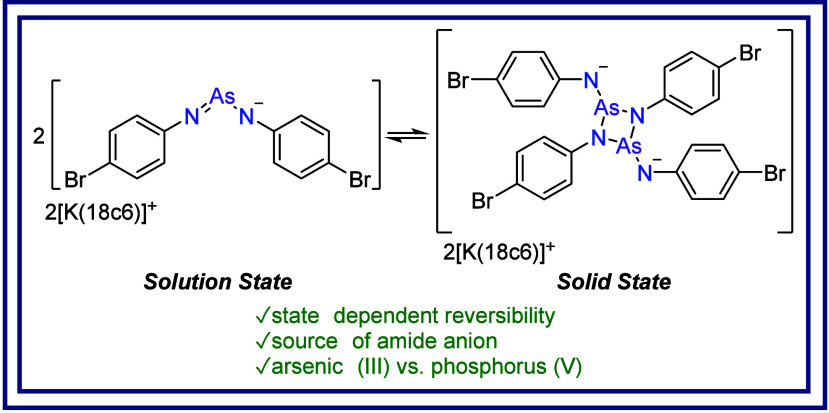

Interest in Zintl cluster chemistry has recently been rekindled, driven by their fascinating bonding motifs and visually striking architectures.^1–6^ Current research efforts have concentrated on exploring their coordination chemistry with transition metals to unlock new physical properties,^7–10^ employing them as catalysts for organic transformations,^11–15^ and leveraging them as precursors for the bottom-up solution-phase synthesis of nanostructures.^16^ To deepen our understanding of these materials, it is essential to investigate their reactivity with common organic substrates.

K_3_As_7_ and K_3_P_7_ represent two well-studied Zintl phases, as they are easily synthesized and in the case of K_3_P_7_ possess the NMR active ^31^P nuclei which enables *in situ* reactivity studies. These phases are known to fragment upon reaction with organic substrates. For example, Goicoechea and co-workers have demonstrated that reaction of K_3_Pn_7_ (Pn = P, As) with alkynes results in the formal transfer of a [Pn_3_]^−^ fragment to yield 1,2,3-tripnictolides,^17–19^ whereas reaction with carbon monoxide leads to formal [P]^−^ transfer to give the [PCO]^−^ anion.^20^ Previously, we have studied the reaction of extracted tetrel-functionalized [Pn_7_] cages from these phases with azides (RN_3_), which were found to undergo insertion of [RN] units into the tetrel-pnictogen bonds of the clusters,^21^ and isolated [K­(DME)_*x*_]_3_[As_7_] with benzyl azide, which was found to yield a 1,2,4-diazarsolide anion.^22^ Here, we study the K_3_Pn_7_ phases directly with aromatic azides, which instead is found to give structures featuring diazadipnictogen rings ({N_2_Pn_2_}) in the solid state. In the case of the arsenic salts, amido­(imino)­arsenide persists in solution and bis­(amido)­diazadiarsenide persists in the solid state. These two compounds can be considered to share a monomer–dimer relationship, whereas for the phosphorus phase, only the dimer is detected in both the solution and solid state. Examples of related amido­(imino)­pnictogenides and their corresponding dimers have been reported (Figure 1),^23–31^ and notably iminochlorophosphanes can alter between monomer and dimer states depending on the steric bulk of auxiliary groups.^32,33^ To the best of our knowledge, this represents the first example where interconversion between the monomer–dimer structure is observed without any structural modifications. Molecules containing such cyclic diazadipnictogen, {Pn_2_N_2_}, fragments can serve as versatile precursors for the synthesis of (poly)­cyclic inorganic and organometallic materials.^34–36^

**Figure 1:**
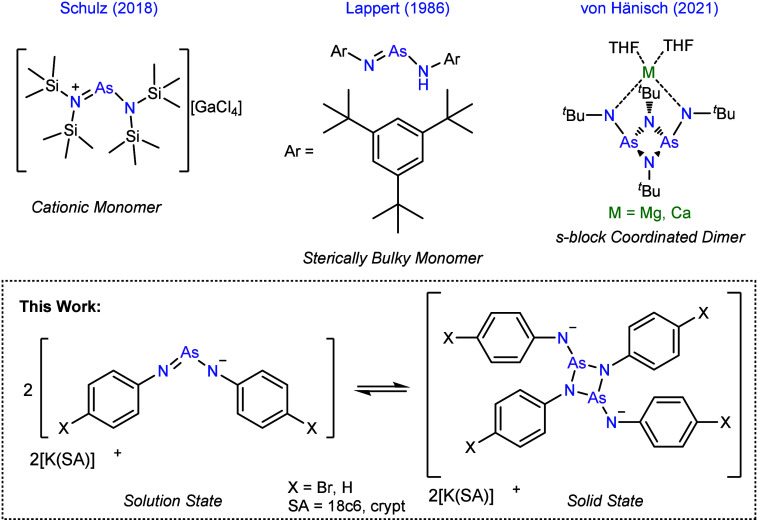
Select examples of molecules featuring {AsN_2_} and {As_2_N_4_} motifs and the monomer–dimer relationship presented in this work.

First, the K_3_As_7_ phase was reacted with 2 equiv of 1-azido-4-bromobenzene (4-BrC_6_H_4_N_3_) in tetrahydrofuran (THF) in the presence of 1,4,7,10,13,16-hexaoxacyclooctadecane (18c6) for 1 h (Figure 2), resulting in evolution of a gas (presumed to be N_2_) and a color change to a red solution with black precipitate. The black precipitates were insoluble in THF and dimethylformamide (DMF) solvents and are presumed to be insoluble polyarsides. We have previously reported that these types of clusters are prone to decomposition upon oxidation to give insoluble polypnictogens.^21^

**Figure 2:**
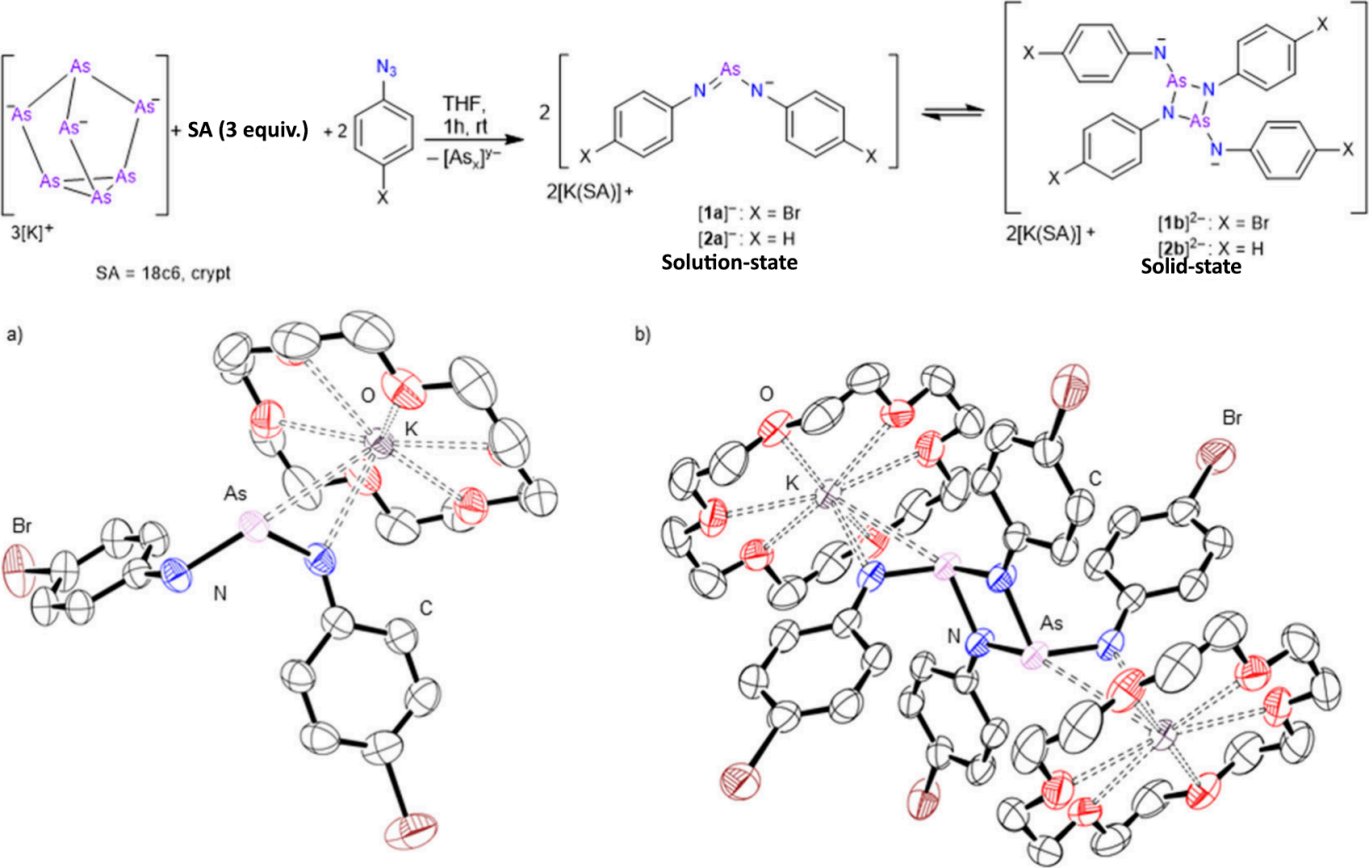
Top: Synthesis and monomer–dimer equilibrium were between [**1a**]^−^ and [**1b**]^2–^. Bottom: Molecular structure of [K­(18c6)]_2_[**1b**]: a) asymmetric unit cell; and b) grown molecular structure. Anisotropic displacement ellipsoids are pictured at 50% probability. Hydrogen atoms and THF molecules have been omitted for clarity. Nitrogen: blue. Carbon: white. Arsenic: plum. Bromine: brown. Oxygen: red. Potassium: violet.

Crystals suitable for analysis by single-crystal X-ray diffraction (SC-XRD) were obtained from the red THF solution at −35 °C and revealed the solution-soluble product to be compound [K­(18c6)]_2_[As_2_(NC_6_H_4_Br)_4_] ([K­(18c6]_2_[**1b**]) in the solid state where each arsenic is in the +3 oxidation state. (Figure 2) Single crystals of [K­(18c6]_2_[**1b**]) could also be grown from acetonitrile, THF, or DMF solutions. The asymmetric unit cell consisted of [K­(18c6)]­[As­(NC_6_H_4_Br)_2_] with one As–N bond being longer (1.917(3) Å) than the other (1.764(3) Å), in line with one nitrogen center being anionic. To our knowledge, there are only two other reports of related dianionic {As_2_N_4_} motifs with s-block metal cations, both described by von Hänisch, whereby the coordinated magnesium or calcium induces large ring strain (Figure 1).^23^ However, similar tetraanionic cage structures have been reported by Wright et al.,^37^ and other related {Pn_4_} rings have also been reported.^38–40^

When the reaction mixture was analyzed by nuclear magnetic resonance (NMR) spectroscopy, only one set of aromatic resonances (δ = 7.18 and 6.57 ppm, ^3^ *J*_H–H_ = 8.8 Hz) was observed, rather than the two that would be expected from the solid-state structure. These data suggest that [K­(18c6)]_2_[**1b**] exists as a dimer in the solid state but as a monomer in solution, [K­(18c6)]­[**1a**] (Figure 2), similar to that presented in the asymmetric unit cell but with additional multiple bond character between arsenic and the neutral nitrogen (Figure 2). Low-temperature NMR studies were attempted at – 40 °C, but no evidence of dimer formation was observed. NMR spectroscopy in a variety of solvents (DMF-*d*_7_, THF-*d*_8_, methanol-d_4_, and acetonitrile-d_3_), both bench-stored and dried, showed only the presence of the monomer in solution. To further validate this hypothesis, ^1^H DOSY NMR studies were undertaken and found to give a diffusion coefficient of 1.4 × 10^–9^ m^2^s^–1^ which gives rise to a predicted molecular weight of 364 g mol^–1^. This predicted mass is within the expected ∼ 15% error of the molecular weight of the monoanionic monomer (412.8 g mol^–1^), and it confirms its presence in solution.^41,42^ Unlike the related species reported by von Hänisch, recrystallization followed by redissolution of this system allows for interconversion between the monomeric and dimeric structures (confirmed by NMR spectroscopy and SC-XRD analysis, respectively).^23^ Further, electrospray ionization mass spectrometry studies show the exclusive presence of a *m*/*z* peak consistent with the monomer in the gas phase. Changing the sequestering agent to 4,7,13,16,21,24-hexaoxa-1,10-diazabicyclo[8.8.8]­hexacosane (crypt) or using azidobenzene as the starting material to make [K­(crypt)]­[**2**] did not have any effect on the monomer–dimer relationship ().

The difference in stability (Gibbs free energy, ΔG) between the monomer and dimer was probed by density functional theory (DFT, ). When studying the gas-phase ΔG of just the anions ([**1b**]^2–^ and 2­[**1a**]^−^), we see that the monomer is ∼ 31 kcal mol^–1^ more stable. However, when calculating the ΔG of the anions solvated by THF, they are almost identical in stability. Further, addition of the 18c6 sequestered potassium cations to the calculations (2­[K­(18c6)]­[**1a**] and [K­(18c6)]_2_[**1b**]) indicates that the dimer is ∼ 6 kcal mol^–1^ more stable. Thus, we postulate that solvation and availability of the cation toward coordination play an important role in whether the dimeric or monomeric form is preferred. It is noteworthy that a related cationic bis­(amino)­arsenium with a [GaCl_4_]^−^ counterion has been reported in exclusively its monomeric form.^31^

To better understand the relationship between [**1a**]^−^ and [**1b**]^2–^, additional DFT investigations were undertaken on both [K­(18c6)]­[**1a**] and [K­(18c6)]_2_[**1b**]. Natural bond occupation analysis of the monomer shows that arsenic has a σ bond to each nitrogen and an additional π bond to one of the nitrogen atoms. Analysis of the calculated Kohn–Sham molecular orbitals shows that both the highest occupied molecular orbital (HOMO) and the lowest unoccupied molecular orbital (LUMO) of the monomer [**1a**]^−^ consist of large p-orbital contributions from the nitrogen atoms (Figure 3). The LUMO also has a significant arsenic p-orbital contribution. These data suggest the potential for [K­(18c6)]­[**1a**] to undergo a [2 + 2] cycloaddition with itself, which would result in [K­(18c6)]_2_[**1b**]. Interestingly, a transition state where lengthening of one of the As–N bonds as the two monomers approach each other can be located with an ΔG energy barrier of ∼ 20 kcal mol^–1^ (Figure 3).

**Figure 3:**
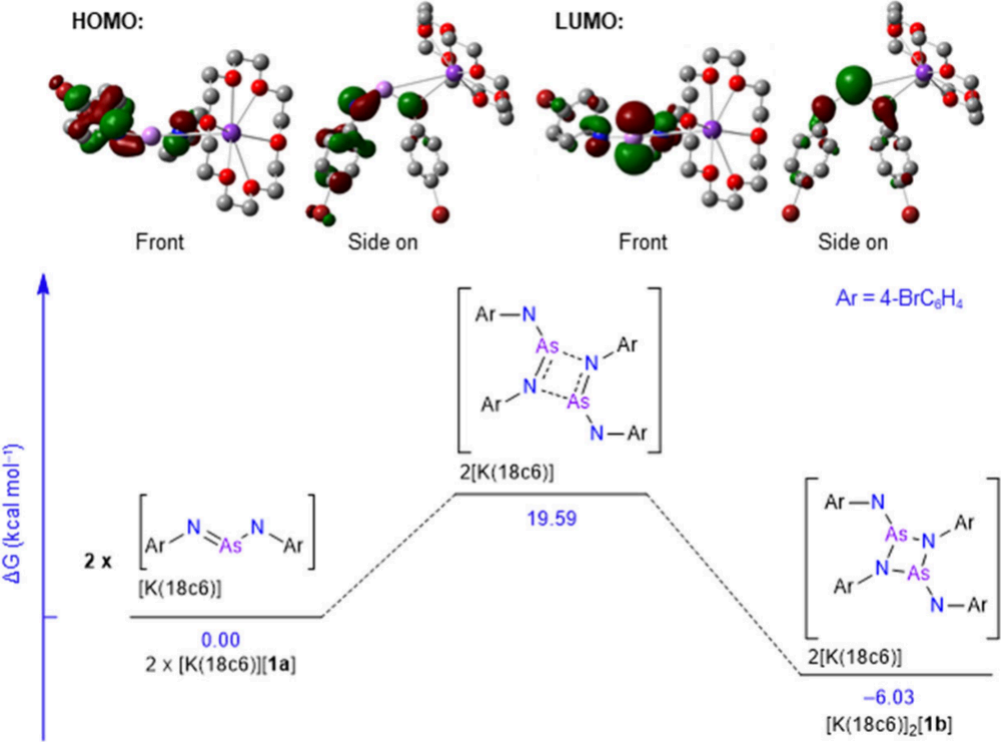
Top: calculated Kohn–Sham molecular orbitals (isovalue = 0.05) of [K­(18c6)]­[**1a**]: Arsenic: light purple. Nitrogen: blue. Carbon: gray. Bromine: brown. Oxygen: red. Potassium: dark purple. Bottom: calculated route of dimerization from 2­[K­(18c6)]­[**1a**] to [K18c6)]_2_[**1b**] via a [2 + 2] cycloaddition.

Compound [K­(18c6)]­[**1a**] appears orange/red in solution; thus, it was investigated by ultraviolet–visible (UV–vis) spectroscopy. Absorption could be seen in the purple/blue region of the spectrum, consistent with this observed color, which was further probed by time-dependent density functional theory and natural transition orbital calculations. The calculations find an absorbance band at 414 nm that consisted of two major transitions, the HOMO to LUMO and HOMO–1 to LUMO (see ). It has previously been reported that four-membered pnictogen containing rings can stabilize diradicals;^15^ thus, [K­(18c6)]­[**1a**] was investigated by cyclic voltammetry studies (acetonitrile solvent, [NBu_4_]­[PF_6_] electrolyte, glassy carbon working electrode, Ag/AgCl leak proof reference electrode, platinum counter electrode) to assess the presence of any reversible redox events. Unfortunately, [K­(18c6)]­[**1a**] cannot be reduced with potentials up to – 2 V, and all oxidation events were irreversible. Attempts were made to oxidize [K­(18c6)]­[**1a**] chemically with N_2_O gas and XeF_2_, but no reaction occurred in either instance.

However, considering that [K­(18c6)]­[**1a**] is prone to [2 + 2] cycloaddition with itself to give the [K­(18c6)]_2_[**1b**], the solution-state reactivity of [K­(18c6)]­[**1a**] toward substrates featuring polarized unsaturated bonds was surveyed. When [K­(18c6)]­[**1a**] was allowed to react with 1 atm of ^13^CO_2_ in a protic solvent (not predried DMF or methanol), a new resonance grew into the ^13^C­{^1^H} NMR spectrum at 159 ppm. Meanwhile, the ^1^H NMR spectrum showed complete consumption of [**1a**]^−^ and conversion to a single product with one set of aromatic resonances detected (δ = 7.62 and 7.16 ppm, ^3^ *J*_H–H_ = 8.8 Hz). A small amount of white solid precipitated out of the reaction mixture, which was partially soluble in CH_3_OH. Subsequent analysis by mass spectrometry showed a *m*/*z* peak in line with [AsO_2_]^−^ with the remaining insoluble solid presumed to be other arsenic oxides. After the reaction mixture was filtered, the solution-soluble product slowly recrystallized, and subsequent SC-XRD studies confirmed the formation of potassium (4-bromophenyl)­carbamate ([K­(18c6)]­[**3**], Figure 4). Reactivity with other substrates featuring C–O multiple bonds, specifically, CO gas, benzophenone, and 4-chlorophenylisocyanate, was also probed, but in all cases, no reaction with [K­(18c6)]­[**1a**] was observed. However, when [K­(18c6)]­[**1a**] was reacted with bench-stored CS_2_ (used as the solvent) the corresponding thiourea derivative *N*,*N*′-bis­(4-bromophenyl)­thiourea (**4**) was formed stoichiometrically, with its structure confirmed by SC-XRD analysis (structure previously reported).^43,44^ Unsurprisingly, mass spectrometry studies into the crude reaction mixture showed the formation of various arsenic sulfides (e.g., *m*/*z* peaks in line with [AsS_2_]^−^, [AsS_3_]^−^, [AsS_5_]^−^, and [AsS_6_]^−^). Reaction of [K­(18c6)]­[**1a**] with excess 4-iodobenzaldehyde (4-IC_6_H_4_CHO) resulted in formation of 2 equiv of the corresponding imine (4-BrC_6_H_4_NCC_6_H_4_-4-I, **5**), in agreement with literature NMR data.^45^ A small amount of insoluble black powder precipitated out of the reaction mixture, presumed to be insoluble polyarsides. These reactions demonstrate that [K­(18c6)]­[**1a**] is prone to amide transfer and loss of arsenic-containing solids, rather than [2 + 2] cycloaddition across the As=N bond.

**Figure 4:**
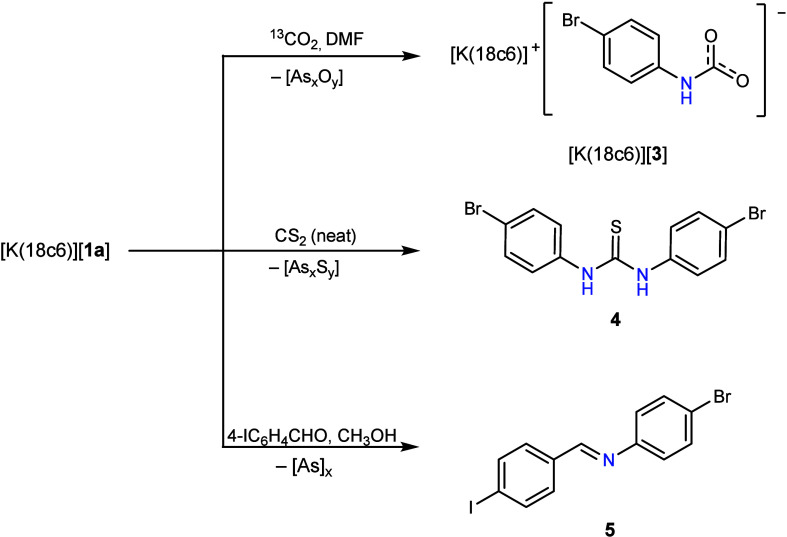
Reactivity studies of [K­(18c6)]­[**1a**].

The K_3_P_7_ phase was also reacted with 4-BrC_6_H_4_N_3_ in an attempt to synthesize the phosphorus derivative of [K­(18c6)]_2_[**1b**] (Figure 5). However, single crystals suitable for SC-XRD analysis from the reaction mixture revealed the formation of [K­(18c6)]_2_[P_2_(NC_6_H_4_Br)_6_] ([K­(18c6)]_2_[**6**]) where each phosphorus is in the +5 oxidation state. Interestingly, [K­(18c6)]_2_[**6**] was found to also exist as what can be considered the dimer of [K­(18c6)]­[(4-BrC_6_H_4_N)_3_P] in the solid state, unlike other tris­(imino)­metaphosphates, reported by Schoeller and co-workers, which exist exclusively as monomers.^46^ The dimeric [K­(18c6)]_2_[**6**] form vs. [K­(18c6)]­[(4-BrC_6_H_4_N)_3_P] is presumably preferred due to the reduced steric bulk of the aryl groups compared to Schoeller’s system (4-bromophenyl vs 2,4,6-tri*tert*-butylphenyl and *tert*-butyl) and more weakly coordinating cation ([K­(18c6)] vs. [Li]). DFT studies were conducted to analyze ΔG between the dimer and proposed monomer form and confirmed the dimeric form [K­(18c6)]_2_[**6**] to be ∼ 12 kcal mol^–1^ more stable. The additional nitrogen groups around each phosphorus compared with [K­(18c6)]_2_[**1b**] can be rationalized by the greater stability of P­(V) vs. As­(V).^47^ The ^31^P NMR spectrum of [K­(18c6)]_2_[**6**] revealed only one singlet resonance at – 24 ppm, while the ^1^H NMR spectrum shows two sets of aromatic signals with a ratio of 2:1. ^1^H DOSY NMR spectroscopy studies revealed a diffusion coefficient of 9.1 × 10^–10^ m^2^ s^–1^ which is indicative of a molecular mass of 972 g mol^–1^, in closer agreement with the mass of the dimeric form [**6**]^2–^ (1082 g mol^–1^), suggesting retention of the dimeric structure in solution. However, electrospray ionization mass spectrometry studies revealed a *m*/*z* value of 557.8409, in line with [K­(18c6)]­[(4-BrC_6_H_4_N)_3_P]·H_2_O ([K­(18c6)]_2_[**6**] in its monomeric form with a coordinated water molecule).

**Figure 5:**
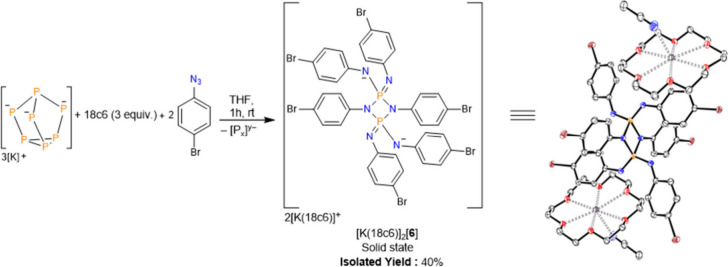
Synthesis and molecular structure of [K­(18c6)]_2_[**6**]. Anisotropic displacement ellipsoids pictured at 50% probability. Hydrogen atoms have been omitted for clarity. Nitrogen: blue. Carbon: white. Phosphorus: orange. Bromine: brown. Oxygen: red. Potassium: violet.

In conclusion, the reaction of K_3_As_7_ and K_3_P_7_ with aromatic azides affords anionic structures featuring {N_2_Pn_2_} rings in the solid state. For the phosphorus system [K­(18c6)]_2_[**6**], the dimeric diazadiphosphenide motif is retained in both the solid and solution states. In contrast, the diazadiarsenide system ([K­(18c6)]_2_[**1b**]) exists in the solid state, but dissolution of this compound in solution leads to the formation of the monomer amido­(imino)­arsenide ([K­(18c6)]­[**1a**]). These species represent a reversible monomer–dimer relationship, where the monomer is thought to undergo a [2 + 2] cycloaddition with itself. However, subsequent reactivity studies show that compound [K­(18c6)]­[**1a**] acts as a stochiometric amide anion donor, yielding carbonate and thiourea derivatives upon exposure to CO_2_ and CS_2_, respectively.

## Supplementary Material





## Notes

General information, experimental procedures, characterization data, and computational detail (PDF)
Coordinates for DFT optimized structures (XYZ)
