# Supplementary material for: Isolation of Potassium Bis(amido)diazadipnictogenide Salts
Source: Organometallics. 2026 Jan 26;45(3):253–8. doi: 10.1021/acs.organomet.5c00493 (PMC12892319; doi:10.1021/acs.organomet.5c00493)
Supplement: Supplementary file 1 [file om5c00493_si_001.pdf]

Supporting Information

Isolation of potassium bis(amido)diazadipnictogenide salts

Reece Lister-Roberts,[1,2] Meera Mehta[1]*

1. Department of Chemistry, University of Oxford, 12 Mansfield Road, Oxford OX1 3TA, U.K.
2. Department of Chemistry, University of Manchester, Oxford Road, Manchester M13 9PL, U.K.

1. Table of Contents

1. Table of Contents 2

2. Methods and Materials 3

2.1 Experimental Considerations 3

2.2 Analytical Considerations 3

2.3 Computational Considerations 5

3. Experimental and Characterisation Data 6

3.1 Synthesis and Characterisation of [K(18c6)]$_2$[**1**] 6

3.2 Synthesis of [K(18c6)]$_2$[**5**] 19

4. Stoichiometric reactivity of [K(18c6)]$_2$[**1**] 25

4.1 Reactivity of [K(18c6)][**1a**] with CO_2 25

4.2 Reactivity of [K(18c6)][**1a**] with CS_2 28

4.3 Reactivity of [K(18c6)]$_2$[**1**] with 4-IC_6H_4CHO 30

5. Crystallographic Tables 31

6. References 32

2. Methods and Materials

2.1 Experimental Considerations

All manipulations were carried out under an inert atmosphere using standard Schlenk-line and glovebox methodology unless specifically stated. All glassware was flame-dried before use.

Innovative Technologies anhydrous engineering solvent purification system was used to obtain dry tetrahydrofuran (THF), dimethylformamide (DMF), diethyl ether, hexane and acetonitrile (CH_3CN). Deuterated chloroform ($CDCl_3$, contains 1% TMS w/w), methanol (MeOD-d_4) and acetonitrile (CD_3CN) were dried and stored over 3 Å molecular sieves. Solvent obtained in this way were subsequently degassed. 1,4,7,10,13,16-hexaoxacyclooctadecane (18c6, Apollo Scientific) was dried by dissolving in dry diethyl ether and storing over 3 Å molecular sieves for a month before filtering and removing the solvent.

The following compounds were purchased from commercial suppliers and used without further purification: 4-iodobenzaldehyde (4-IC_6H_4CHO, Sigma-Aldrich), CS_2 (Sigma-Aldrich), $^{13}CO_2$ (CK Isotopes), ^{t}butyl nitrite (Apollo Scientific), tetramethylsilyl azide (Sigma-Aldrich), Nitrous oxide (CK Isotopes), Xenon Difluoride (Sigma-Aldrich), and 4,7,13,16,21,24-Hexaoxa-1,10-diazabicyclo[8.8.8]hexacosane (crypt; Sigma-Aldrich).

4-Bromoaniline and aniline were purchased from Sigma-Aldrich and purified by vacuum distillation at 130°C.

1-azido-4-bromobenzene, azidobenzene, K_3As_7, and K_3P_7 were prepared according to literature procedures.[1,2]

2.2 Analytical Considerations

NMR spectroscopy. ^1H, $^{13}C\{^1H\}$, $^{31}P\{^1H\}$ NMR spectra were recorded on a Bruker AVIII 400 spectrometer (operating frequencies: 399.78 MHz, 100.53 MHz and 149.14 MHz for ^1H, ^{13}C, and ^{31}P respectively) unless otherwise stated. Variable temperature ^1H and ^1H Diffusion-Ordered Spectroscopy was carried out on a Bruker AVIII 500 spectrometer (operating frequency: 499.94 MHz. ^1H and ^{13}C chemical shifts were internally referenced to the residual solvent resonances (CD_3CN: ^1H δ = 1.94 ppm, $^{13}C\{^1H\}$ δ = 1.32 ppm, DMF-d_7: ^1H δ = 8.03, $^{13}C\{^1H\}$ δ = 163.15, MeOD-d_4 ^1H δ = 4.87 ppm, $CDCl_3$: ^1H δ = 7.26 ppm. ^{31}P chemical shifts were externally referenced to H_3PO_4. NMR samples were prepared under an inert nitrogen atmosphere in a 5 mm J Young NMR tube where dry conditions are stated. All NMR data was analyzed using MestReNova V15.1.0.

Elemental Analysis. Elemental analysis was carried out by London Metropolitan University using a ThermoFlash 2000.

Mass spectrometry. Mass spectrometry samples were analyzed by the mass spectrometry service at the University of Oxford using an electrospray ionization (ESI) equipped Waters RDa bench-top time of flight mass spectrometer. Samples were prepared under a nitrogen atmosphere and directly injected into the ionization source of the mass spectrometer.

Infrared spectroscopy. ATR-IR spectra were recorded on microcrystalline powders using a Bruker Alpha II under an inert atmosphere.

Cyclic Voltammetry. Cyclic voltammetry (CV) was carried out in the glovebox under inert conditions with EMStat4s. Electrodes: Working – glassy carbon. Counter – platinum wire. Reference – Ag /AgCl (leak proof).

Ultraviolet–visible spectroscopy. Ultraviolet-visible (UV-Vis) electronic absorption spectra were recorded on a Mettler Toledo UV5Bio spectrophotometer using 10 mm path length quartz J Young cuvettes.

X-ray diffraction studies. X-ray diffraction data was collected for compounds $[K(18c6)]_2$[**1b**], $[K(crypt)]_2$[**1b**], **4**, and $[K(18c6)]_2$[**6**] on a dual source Rigaku XtaLAB Synergy-DW VHF equipped with a PhotonJet-R dual wavelength rotating anode and HyPix-Arc 150° detector at 100K. X-ray diffraction data for [K(18c6)][**3**] was collected an Oxford Diffraction Supernova dual-source diffractometer at 150K using Cu Kα (1.54184 Å) radiation equipped with a 135 mm Atlas CCD area detector. X-ray diffraction data was collected for [K(crypt)][**2**] on a 100 K dual source Rigaku FR-X rotating anode diffractometer with a Hypix-6000HE detector and an Oxford Cryosystems nitrogen flow gas system. X-ray data was collected using CrysAlisPro software.[3]

Crystal structure determination and refinements. X-ray data was processed and reduced using CrysAlisPro. Absorption correction was performed using empirical methods (SCALE3 ABSPACK) based upon symmetry-equivalent reflections combined with measurements at different azimuthal angles. The crystal structure was solved and refined against all F2 values using the SHELX and Olex2 suite of programmes.[4,5] All atoms were refined anisotropically. Hydrogen atoms were placed in calculated positions and refined using idealized geometries and assigned fixed isotropic displacement parameters.

SIMU, SADI, and RIGU commands were used to model disorder in the THF molecules in $[K(18c6)]_2$[**1b**].

SIMU and SADI restraints were used to model disorder in the 18c6 and DMF units of [K(18c6)][**3**].

Crystallographic data have been deposited with the CCDC (CCDC 2515311 – 2515314, 2522284, and 2523832.

2.3 Computational Considerations

DFT geometry optimizations and frequency calculations were carried out using the Gaussian 09 package, revision D.01.[6–9] All calculations were carried out using B3LYP functional and the Ahlrichs Def2 basis set of polarized triple-ζ quality (Def2-TZVP).[10–12] Geometry optimizations were performed, with default settings, starting from crystallographic coordinates. Optimizations were carried out on the gas phase anions ([**1a**]$^{-}$, [**1b**]$^{2-}$ and [**6**]$^{2-}$) and also with a solvent model (smd = THF). Further optimizations and single point calculations were carried out including the cation and sequestering agents ([K(18c6)][**1a**], [K(18c6)]$_2$[**1b**], [K(18c6)]$_2$[**6**], and [K(18c6)][P(NC_6H_4Br)$_3$]). Analysis of the harmonic vibrational frequencies confirmed the optimized geometries as energetic minima. Calculated IR spectra were generated in GaussView 5.0 using the calculated vibrational frequencies. Where stated, solvent environment was modelled using the smd method, with parameters appropriate to THF.[13] Natural bond orbital (NBO) and natural localized molecular orbital (NLMO) calculations were carried out using NBO 7.0 program.

100 state, full TD-DFT calculations and subsequent NTO calculations were performed using the Gaussian 9 package. The coordinates were taken from the optimized gas phase calculation and not re-optimized.

Plots of the Kohn-Sham and Natural Transition Orbitals were obtained from the Gaussian fchk file and plotted in GaussView 5.0.

Transition state calculations were carried out using QST3 in Gaussian 9 starting from the optimized structures for the solvated monomer and dimer (smd=THF, [K(18c6)][**1a**] and [K(18c6)]$_2$[**1b**]). Analysis of the harmonic vibrational frequencies confirmed only one imaginary frequency in line with a transition state.

All geometry optimized coordinates are supplied as a separate .xyz file.

3. Experimental and Characterisation Data

3.1 Synthesis and Characterisation of [K(18c6)]$_2$[**1**]

In the glovebox, K_3As_7 (286 mg, 0.44 mmol, 1 equiv.) and 18c6 (352 mg, 1.34 mmol, 3 equiv.) were suspended in 3 mL of THF in a vial. 1-Azido-4-bromobenzene (174.3 mg, 0.88 mmol, 2 equiv.) was added in four portions over 30 minutes, and the solution was stirred for a further 30 minutes. The solution was then filtered and placed in the freezer, resulting in formation of a white crystalline precipitate. The solid was then filtered and washed with THF (2 mL) before drying under vacuum. The solid was then dissolved in MeCN (5mL) and left for 30 minutes before filtering and drying under reduced pressure, yielding [K(18c6)]$_2$[**1b**]. Crystals suitable for SC-XRD analysis were grown from a concentrated THF solution in the dark at –35°C or from a concentrated solution under normal conditions (crystals form inside NMR tube above 10mg per 0.5 mL concentration, without the exclusion of light).

Isolated Yield: 220 mg (68%)

^{1}H NMR (CD_3CN): δ = 7.18 (d, $^3J_{H\text{-}H}$ = 8.8 Hz, 2H, Ar*H*), 6.57 (d, $^3J_{H\text{-}H}$ = 8.8 Hz, 2H, Ar*H*), 3.55 (s, 24H, 18c6) ppm.

^{13}C{^{1}H} NMR (CD_3CN): δ = 148.1 (s, Ar*C*), 132.1 (s, Ar*C*), 116.7 (s, Ar*C*), 108.1 (s, Ar*C*), 70.4 (s, 18c6) ppm.

Mass Spectrometry [$C_{12}H_8AsBr_2N_2+H_2O$]$^-$: Calculated: 432.8360 Found: 432.8284

Elemental Analysis (CHN, %, [K(18c6)]$_2$[1].CH_3CN): Expected C: 40.67. H: 4.51. N: 4.74. Found C: 40.45. H: 4.85. N: 4.72

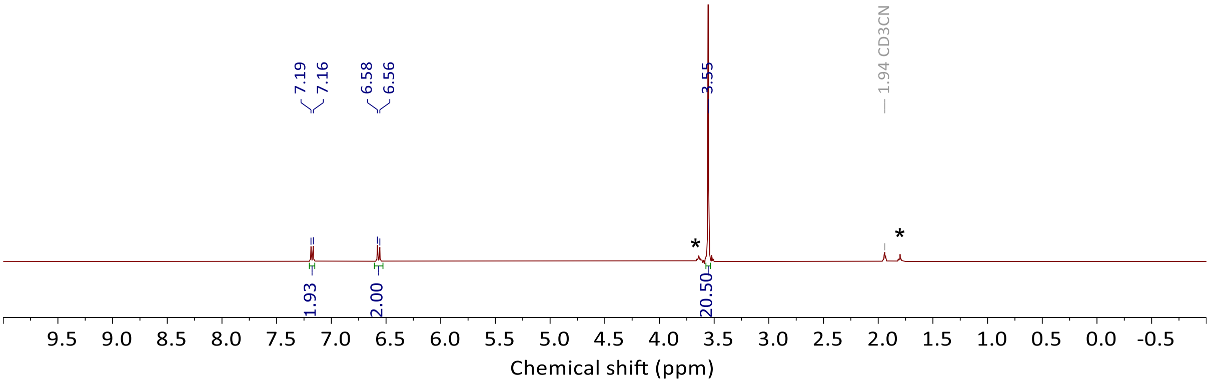

Figure S1: ^{1}H NMR (CD_3CN) of [K(18c6)][**1a**] with coordinated THF marked with an asterisk.

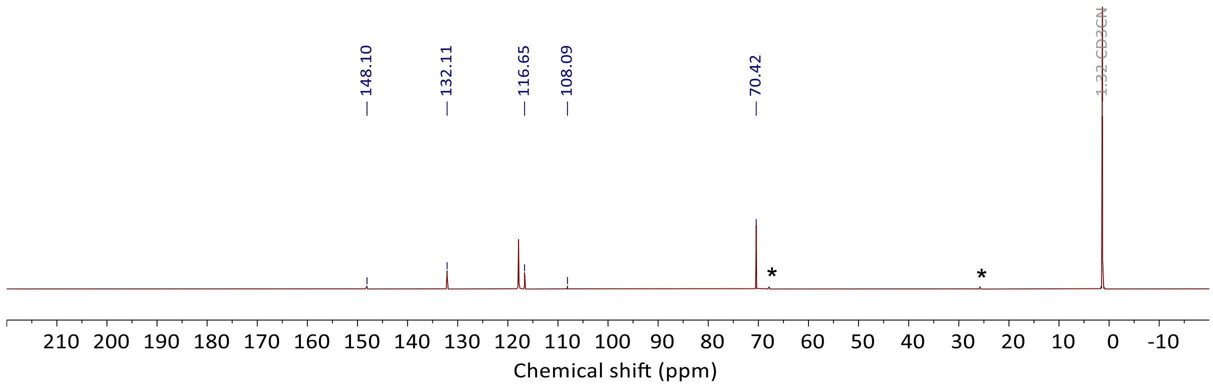

Figure S2: $^{13}C\{^1H\}$ NMR (CD_3CN) of [K(18c6)][**1a**] with coordinated THF marked with an asterisk.

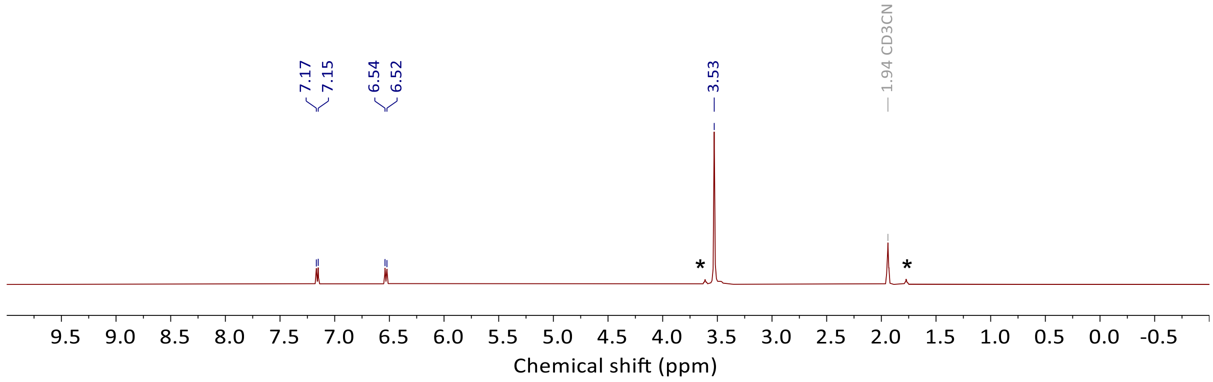

Figure S3: ^1H NMR (CD_3CN) of [K(18c6)][**1a**] at –35 °C with coordinated THF marked with an asterisk.

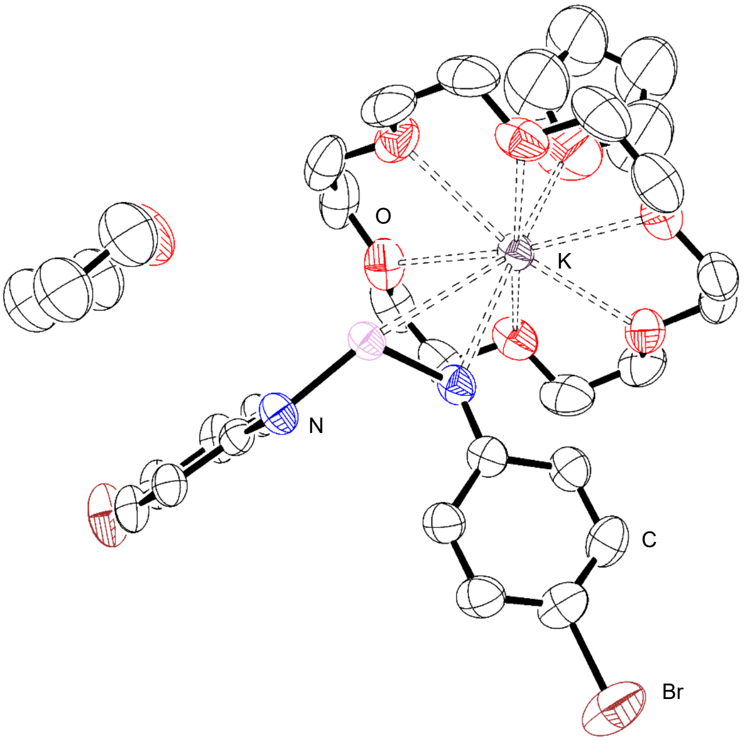

Figure S4: Molecular structure of [K(18c6)]$_2$[**1b**] (asymmetric unit cell). Anisotropic displacement ellipsoids pictured at 50% probability. Anisotropic displacement ellipsoids pictured at 50% probability. Hydrogen atoms omitted for clarity. Nitrogen: blue. Carbon: white. Bromine: brown. Arsenic: plum. Oxygen: red. Potassium: violet.

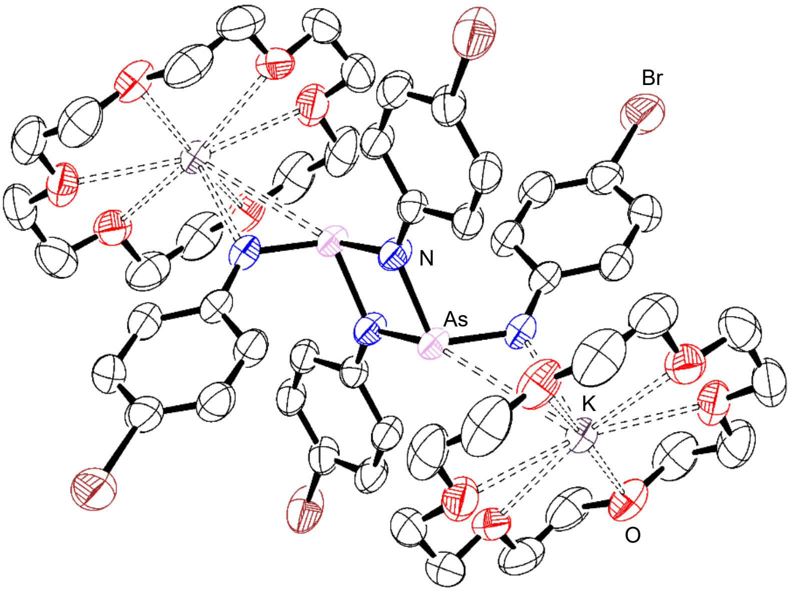

Figure S5: Molecular structure of [K(18c6)]$_2$[**1b**] (fragment grown). Anisotropic displacement ellipsoids pictured at 50% probability. Anisotropic displacement ellipsoids pictured at 50% probability. Hydrogen atoms and THF molecules omitted for clarity. Nitrogen: blue. Carbon: white. Bromine: brown. Arsenic: plum. Oxygen: red. Potassium: violet.

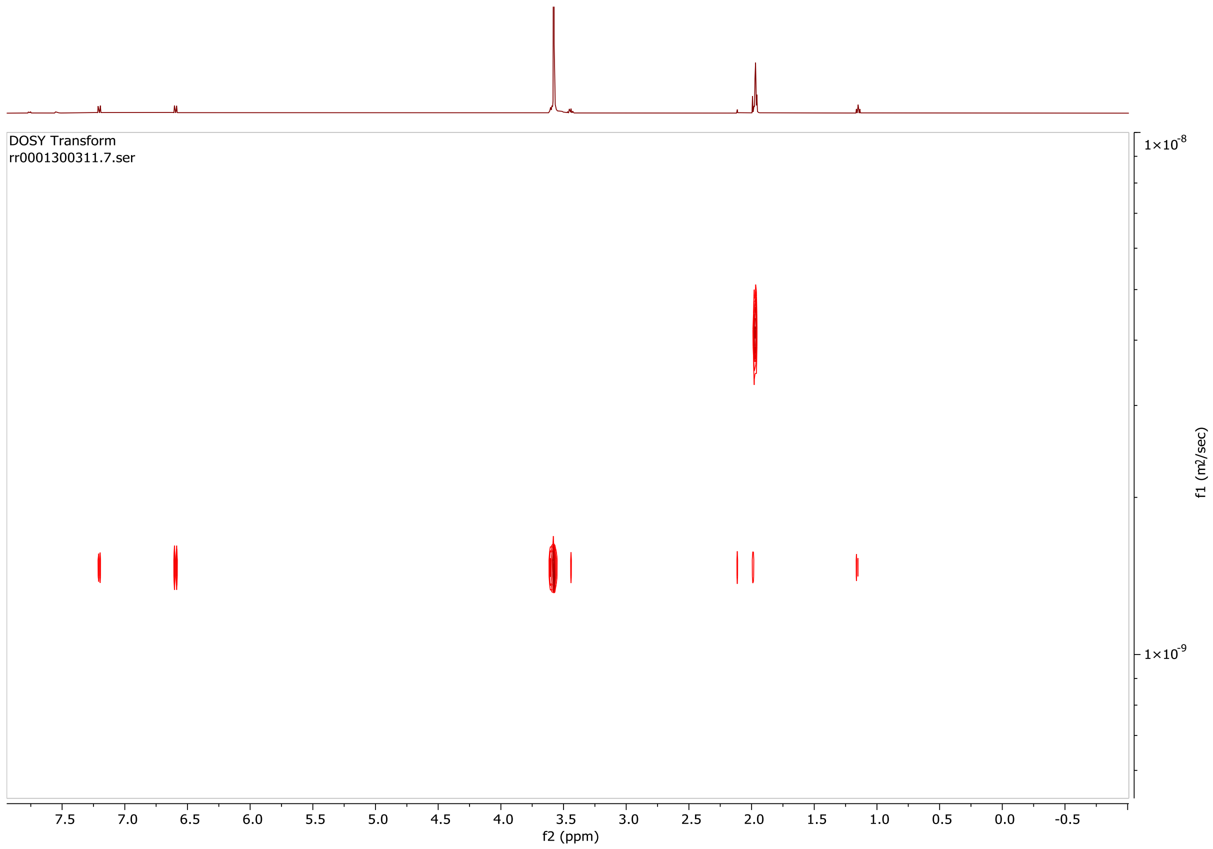

Figure S6: ^{1}H DOSY NMR (CD_3CN) spectrum of [K(18c6)][**1a**].

Table S1: Difference in free enthalpies (ΔG) of [K(18c6)][**1a**] and $[K(18c6)]_2$[**1b**].

Calculation Conditions	Difference in ΔG (kcal mol^{-1}, dimer – monomer)
Gas Phase Anion	30.93
Solvated Anion (THF)	–0.64
Solvated with [K(18c6)]	–6.03

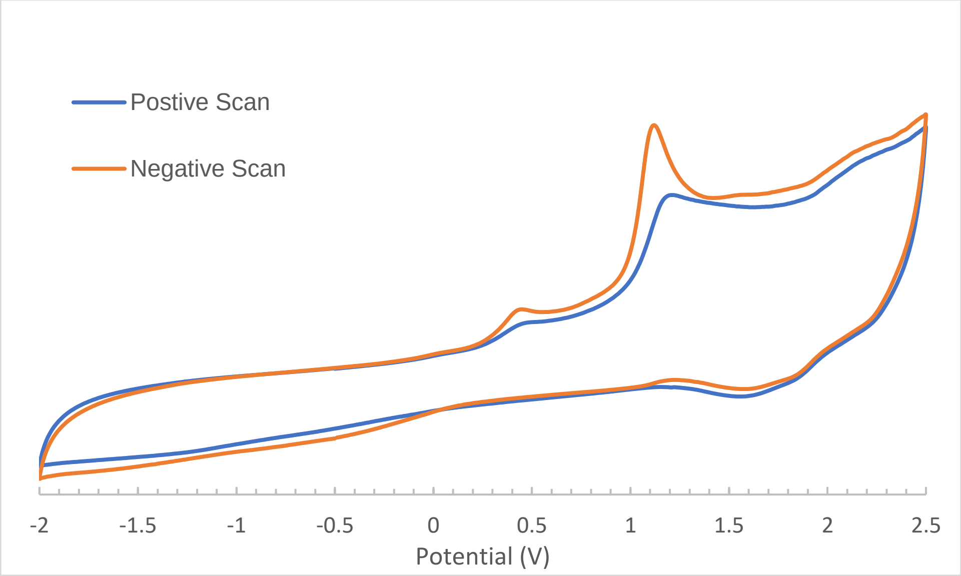

Figure S7: Cyclic voltammetry of [K(18c6)][**1a**] at 3 mM in MeCN with [nBu_4N][PF_6] electrolyte at 0.1 V/s starting at –0.5 V and scanning independently in the positive direction first (blue trace) and the negative direction first (orange trace). Glassy carbon working electrode, platinum counter electrode and leak-proof Ag/AgCl reference electrode were used.

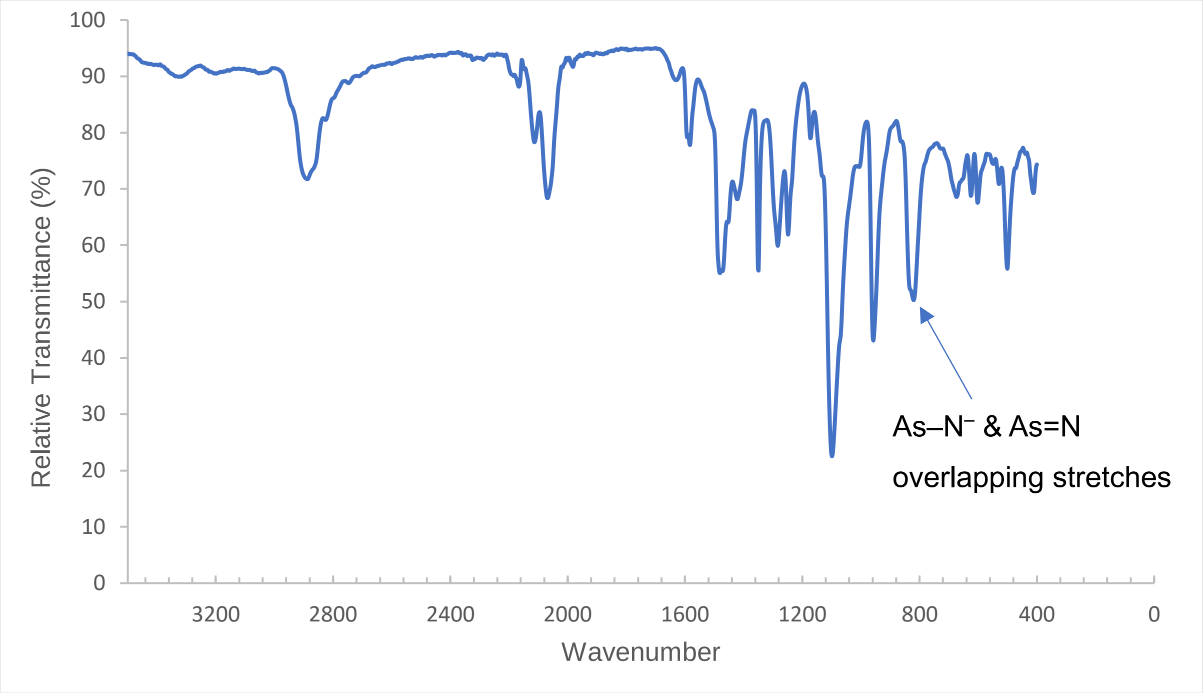

Figure S8: Experimental infrared spectrum of $[K(18c6)]_2$[**1b**].

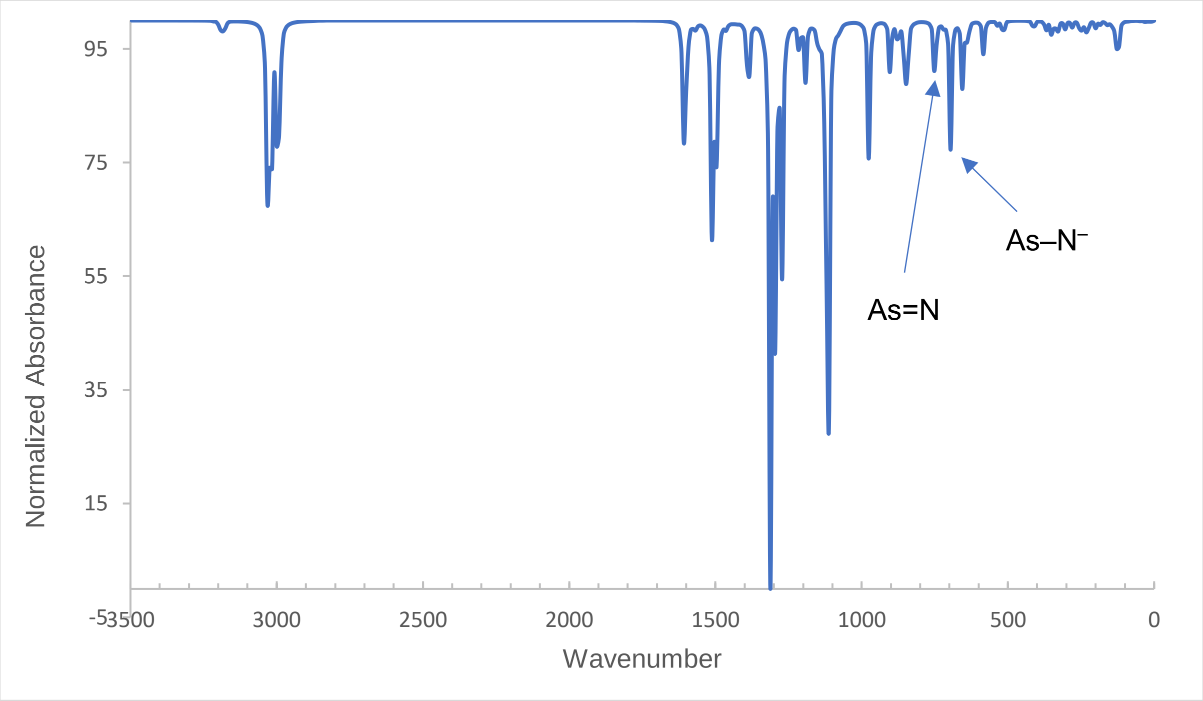

Figure S9: Calculated infrared spectrum of $[K(18c6)]_2$[**1b**].

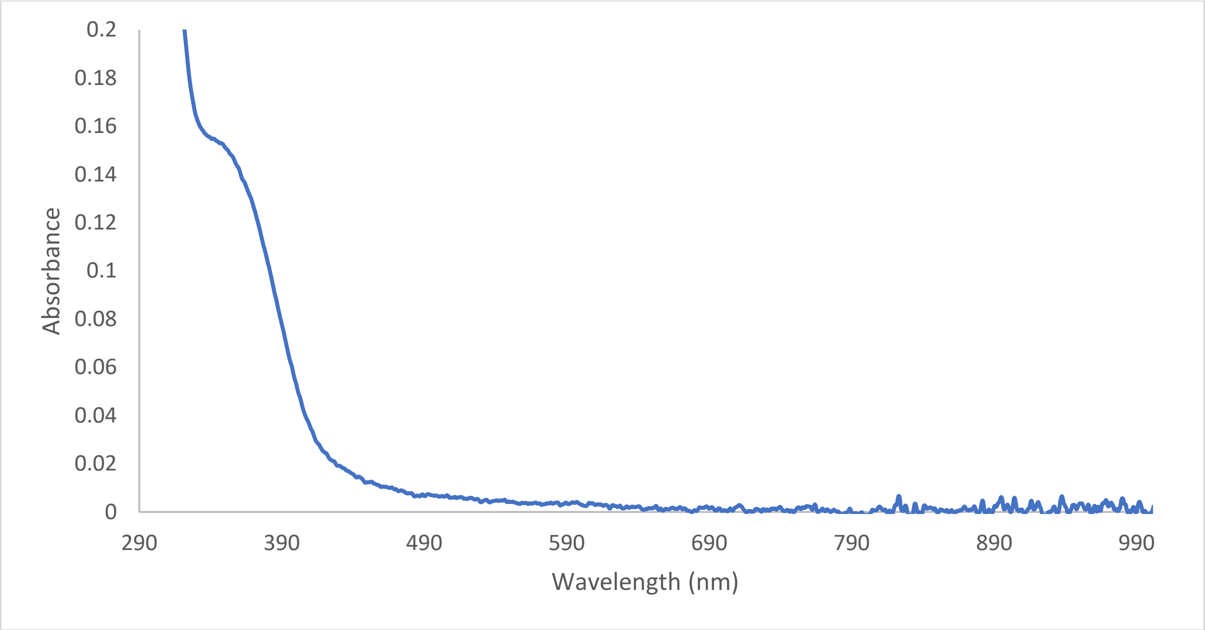

Figure S10: UV-Vis spectrum of [K(18c6)][**1a**] (0.05mM in MeCN).

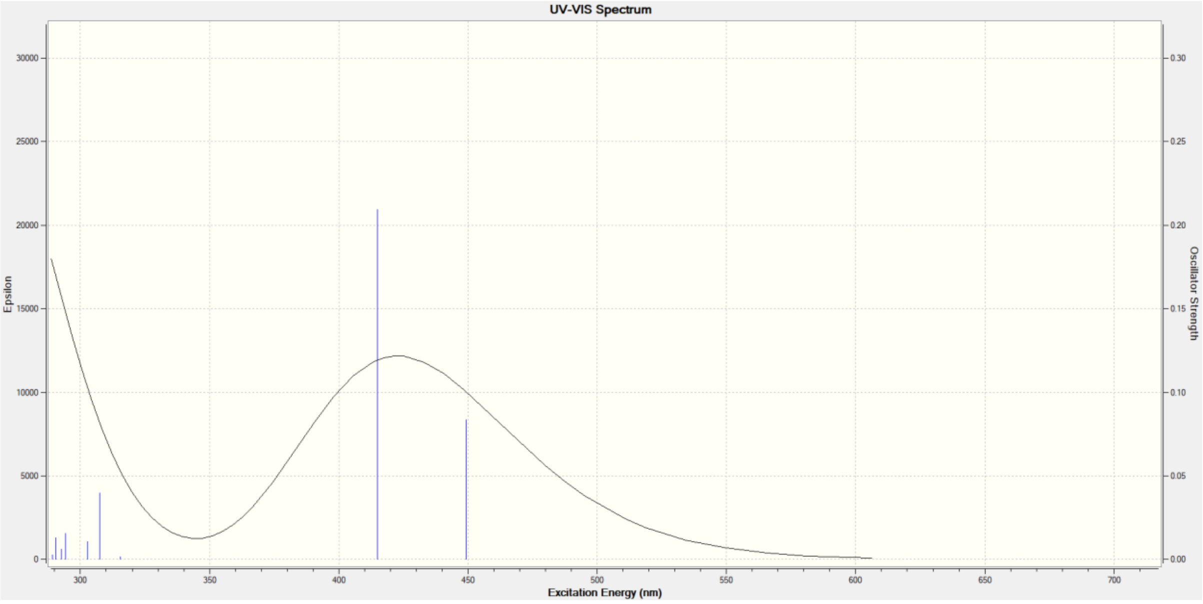

Figure S11: Calculated UV-Vis spectrum of [K(18c6)][**1a**]

Table S2: Description of key transition at 414.9 nm from NTO calculation of [K(18c6)][**1a**].

Orbitals	NTO Occupancy	Kohn-Sham Orbitals
HOMO to LUMO	0.57	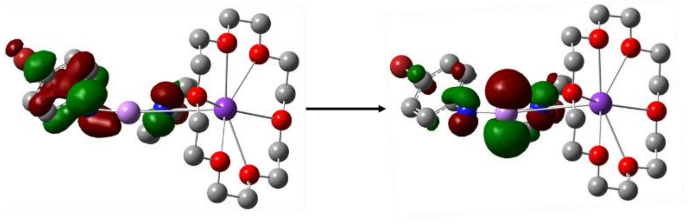
HOMO-1 to LUMO	0.40	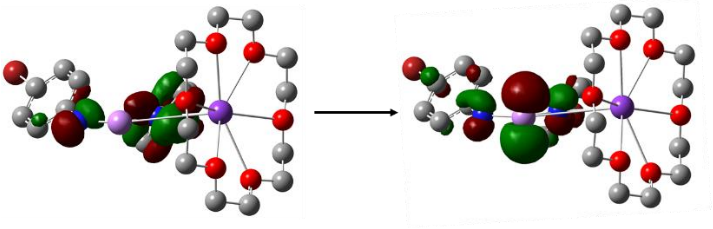

3.2 Synthesis and Characterisation of [K(crypt)][1]

In the glovebox, K_3As_7 (143 mg, 0.22 mmol, 1 equiv.) and crypt (248 mg, 0.66 mmol, 3 equiv.) were suspended in 3 mL of THF in a vial. 1-Azido-4-bromobenzene (87.2 mg, 0.44 mmol, 2 equiv.) was added in five portions over 1 hour, and the solution was stirred for a further 30 minutes. The solution was then filtered and diethyl ether added causing a precipitate to form. The precipitate was filtered and washed with diethyl ether before drying under reduced pressure. The crude product dissolved in 1 mL of THF and left overnight at –35°C yielding $[K(crypt)]_2$[**1b**] as a crystalline solid. Crystals suitable for SC-XRD analysis were grown from a vapor diffusion of hexane into a concentrated THF solution at –35°C.

Isolated Yield: 60 mg (24 %)

^{1}H NMR (CD_3CN): δ = 7.18 (d, $^3J_{H\text{-}H}$ = 8.8 Hz, 2H, Ar*H*), 6.57 (d, $^3J_{H\text{-}H}$ = 8.8 Hz, 2H, Ar*H*), 3.56 (s, 12H, crypt), 3.51 (m, 12H, crypt), 2.52 (m, 12H, crypt) ppm.

$^{13}C\{^1H\}$ NMR (CD_3CN): δ = 133.6 (s, Ar*C*), 117.2 (s, Ar*C*), 71.2 (s, crypt), 68.4 (s, crypt), 54.7 (s, crypt) ppm.

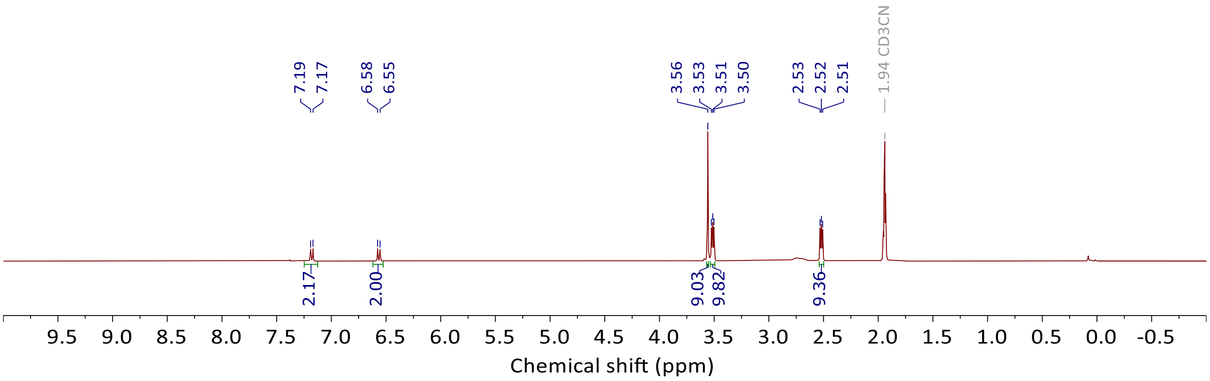

Figure S12: ^{1}H NMR (CD_3CN) of [K(crypt)][**1a**]

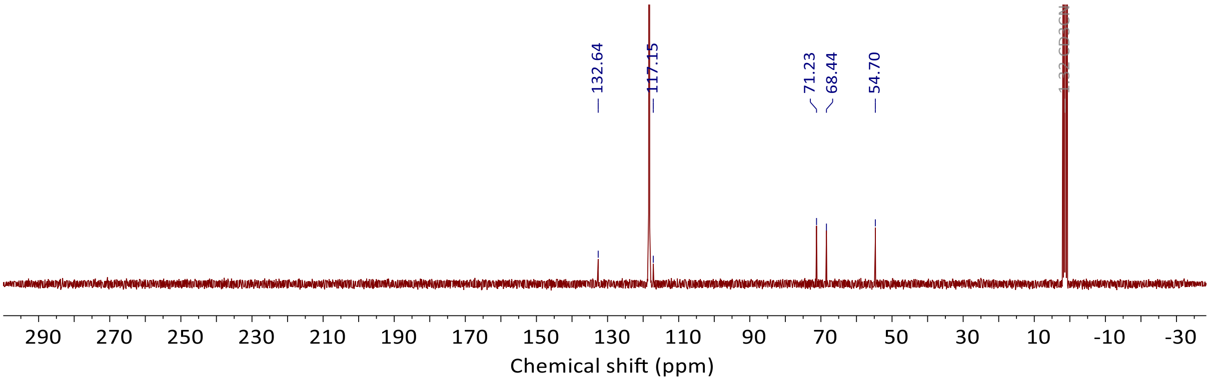

Figure S13: $^{13}C\{^{1}H\}$ NMR (CD_3CN) of [K(crypt)][**1a**].

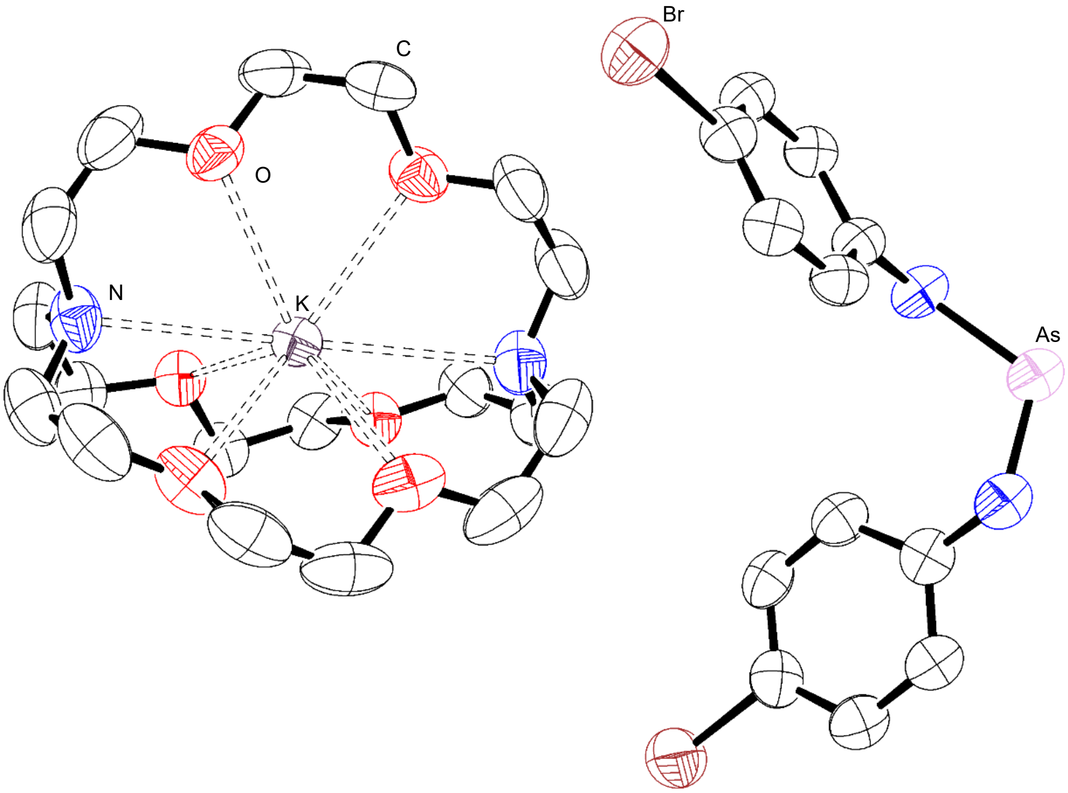

Figure S14: Molecular structure of $[K(crypt)]_2$[**1b**] (asymmetric unit cell). Anisotropic displacement ellipsoids pictured at 50% probability. Anisotropic displacement ellipsoids pictured at 50% probability. Hydrogen atoms omitted for clarity. Nitrogen: blue. Carbon: white. Bromine: brown. Arsenic: plum. Oxygen: red. Potassium: violet.

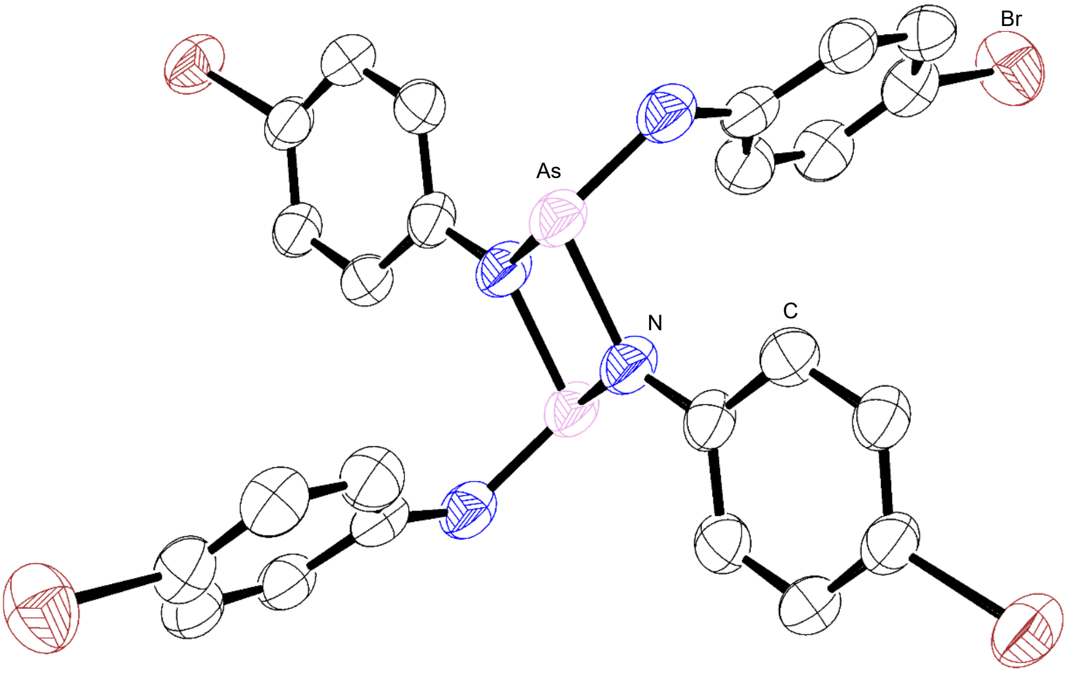

Figure S15: Molecular structure of [K(crypt)]$_2$[**1b**] (fragment grown). Anisotropic displacement ellipsoids pictured at 50% probability. Anisotropic displacement ellipsoids pictured at 50% probability. Hydrogen atoms and [K(crypt)] molecules omitted for clarity. Nitrogen: blue. Carbon: white. Bromine: brown. Arsenic: plum. Oxygen: red. Potassium: violet.

3.3 Synthesis and Characterization of [K(crypt)]$_2$[**2**]

In the glovebox, K_3As_7 (143 mg, 0.22 mmol, 1 equiv.) and crypt (248 mg, 0.66 mmol, 3 equiv.) were suspended in 3 mL of THF in a vial. Azidobenzene (52.4 mg, 0.44 mmol, 2 equiv.) was added in five portions over 1 hour, and the solution was stirred for a further 30 minutes. The solution was then filtered and diethyl ether added causing a precipitate to form. The precipitate was filtered and washed with diethyl ether before drying under reduced pressure yielding [K(crypt)]$_2$[**2**] as a beige solid. Crystals suitable for SC-XRD analysis were grown from a concentrated THF solution at –35°C.

Isolated Yield: 84 mg (28 %)

^{1}H NMR (CD_3CN): δ = 7.10 (t, $^3J_{H\text{-}H}$ = 8.8 Hz, 2H, Ar*H*), 6.65 (m, 3H, Ar*H*), 3.56 (s, 12H, crypt), 3.51 (m, 12H, crypt), 2.52 (m, 12H, crypt) ppm.

^{13}C{^{1}H} NMR (CD_3CN): δ = 148.6 (s, Ar*C*), 129.6 (s, Ar*C*), 117.6 (s, Ar*C*), 114.9 (s, Ar*C*), 70.8 (s, crypt), 68.0 (s, crypt), 54.3 (s, crypt) ppm.

Mass Spectrometry [$C_{12}H_{11}AsN_2$]$^-$: Calculated: 258.0143 Found: 258.0142

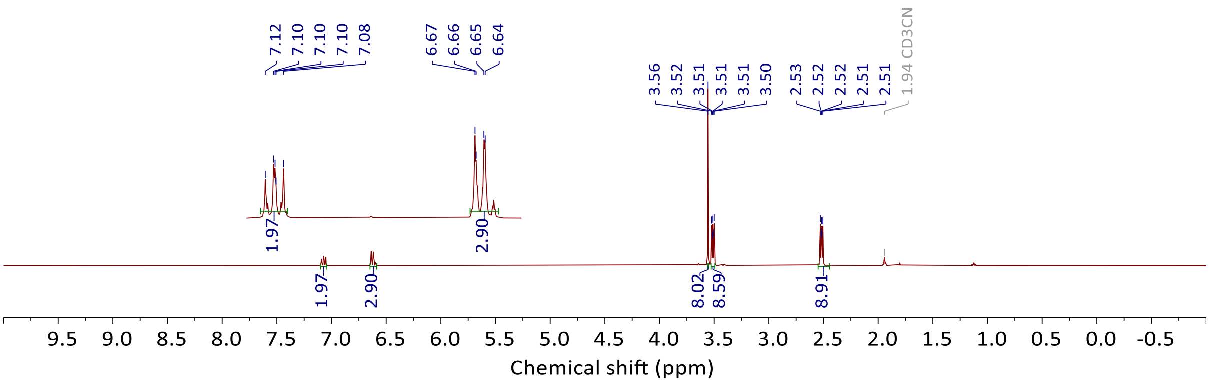

Figure S16: ^{1}H NMR (CD_3CN) spectrum of [K(crypt)]$_2$[**2a**] with an inset zoomed in on the aromatic region.

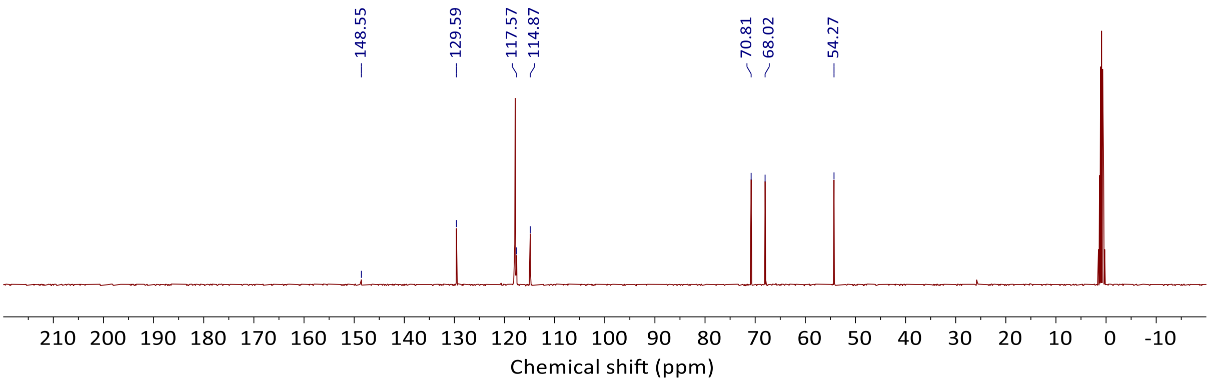

Figure S17: ^{13}C{^{1}H} NMR (CD_3CN) spectrum of [K(crypt)]$_2$[**2a**].

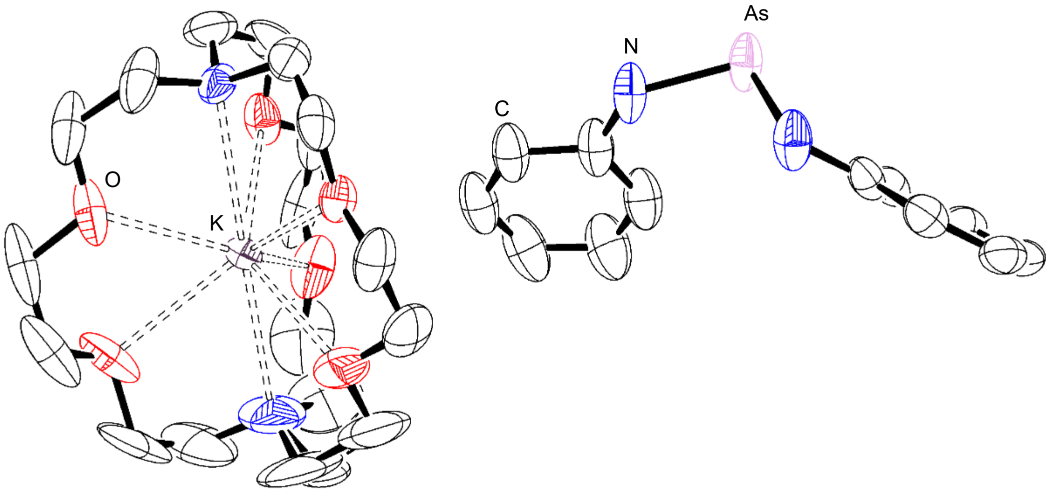

Figure S18: Molecular structure of [K(18c6)]$_2$[**2b**] (asymmetric unit cell). Anisotropic displacement ellipsoids pictured at 50% probability. Anisotropic displacement ellipsoids pictured at 50% probability. Hydrogen atoms omitted for clarity. Nitrogen: blue. Carbon: white. Arsenic: plum. Oxygen: red. Potassium: violet.

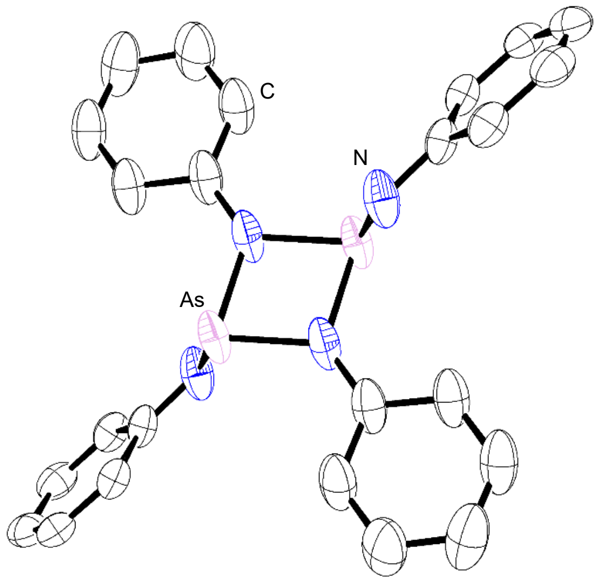

Figure S19: Molecular structure of [K(18c6)]$_2$[**2b**] (fragment grown). Anisotropic displacement ellipsoids pictured at 50% probability. Anisotropic displacement ellipsoids pictured at 50% probability. Hydrogen atoms and [K(crypt)] molecules omitted for clarity. Nitrogen: blue. Carbon: white. Arsenic: plum. Oxygen: red. Potassium: violet.

3.4 Synthesis of [K(18c6)]$_2$[**6**]

In a vial in the glovebox, K_3P_7 (147 mg, 0.44 mmol, 1 equiv.) and 18c6 (352 mg, 1.34 mmol, 3 equiv.) were suspended in THF and 1-azido-4-bromobenzene (261.4 mg, 1.32 mmol, 3 equiv.) was added in 6 portions over 30 minutes and then stirred for a further 30 minutes. The solution was then filtered and hexane added causing a precipitate to form. The oily, red solid that forms was dried under vacuum before washing with THF (2 x 5mL) leaving to settle for 5 minutes each time before filtering. The remaining solid was then dried giving [K(18c6)]$_2$[**6**] as a white solid. Crystals suitable for SC-XRD analysis were obtained from a concentrated CD_3CN solution.

Isolated Yield: 450 mg (40%)

^{1}H NMR (CD_3CN): δ = 7.22 (d, $^3J_{H-H}$=8.8 Hz, 4H), 7.15 (d, $^3J_{H-H}$=8.8 Hz, 2H), 7.02 (d, $^3J_{H-H}$=8.8 Hz, 2H), 6.99 (d, $^3J_{H-H}$=8.8 Hz, 4H), 3,56 (s, 24H, 18c6) ppm.

^{13}C{^{1}H} NMR (CD_3CN): δ = 132.48 (s, Ar*C*), 131.57 (s, Ar*C*), 126.30 (s, Ar*C*), 120.14 (s, Ar*C*), 70.93 (s, 18c6) ppm.

^{31}P NMR (CD_3CN): δ = –24.57 (s) ppm.

Mass Spectrometry [$C_{18}H_{12}Br_3N_3P+H_2O]^-$: Calculated: 557.8409 Found: 557.8406

Elemental Analysis (CHN, %, [K(18c6)]$_2$[6]): Expected C: 42.67. H: 4.30. N: 4.98. Found C: 42.65. H: 4.53. N: 4.43.

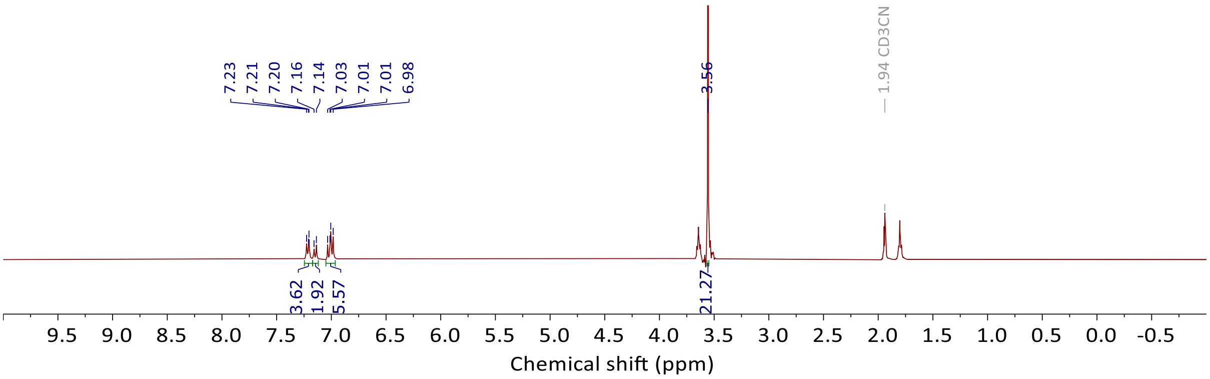

Figure S20: ^{1}H NMR (CD_3CN) spectrum of [K(18c6)]$_2$[**6**].

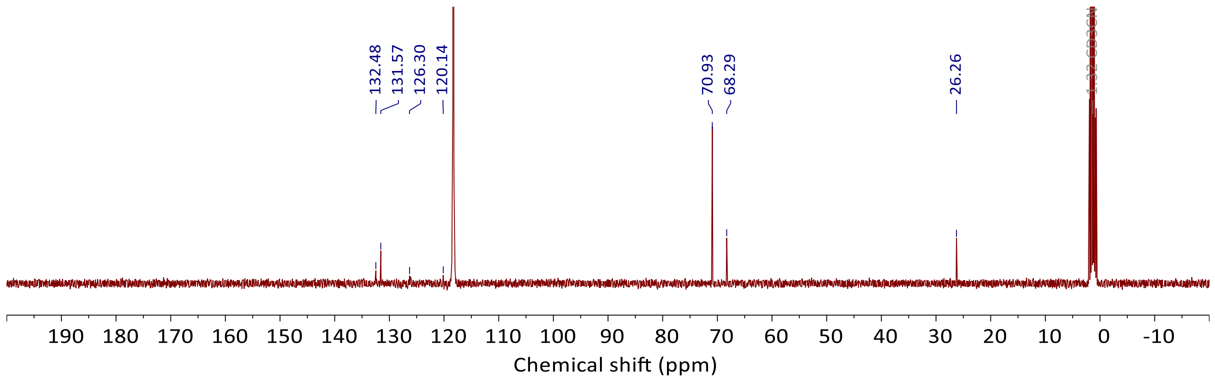

Figure S21: $^{13}C\{^1H\}$ NMR (CD_3CN) spectrum of $[K(18c6)]_2$[**6**].

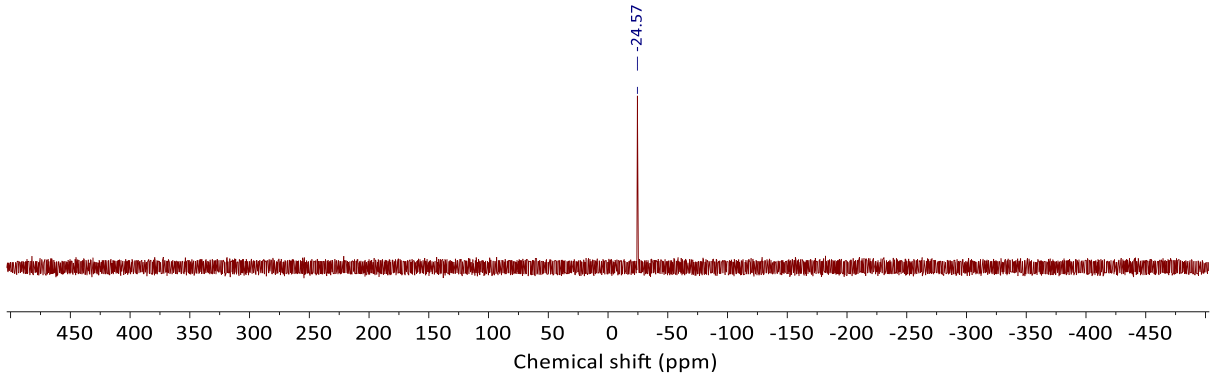

Figure S22: ^{31}P NMR (CD_3CN) spectrum of $[K(18c6)]_2$[**6**].

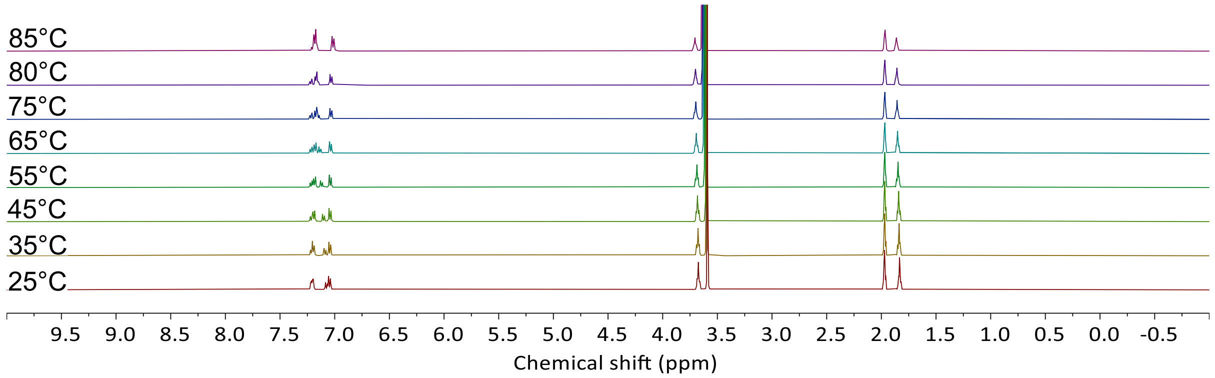

Figure S23: Stacked variable temperature ^1H NMR (CD_3CN) spectrum of $[K(18c6)]_2$[**6**].

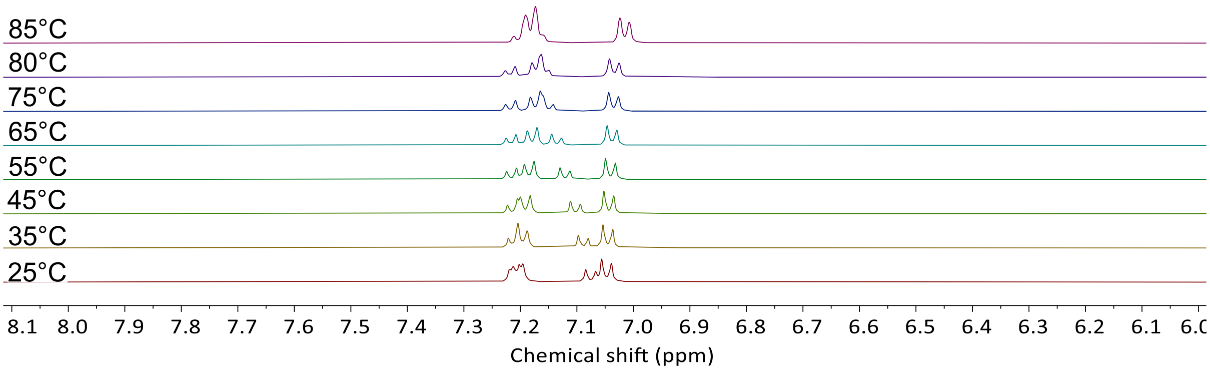

Figure S24: Zoomed in stacked variable temperature ^1H NMR (CD_3CN) spectrum of [K(18c6)]$_2$[**6**].

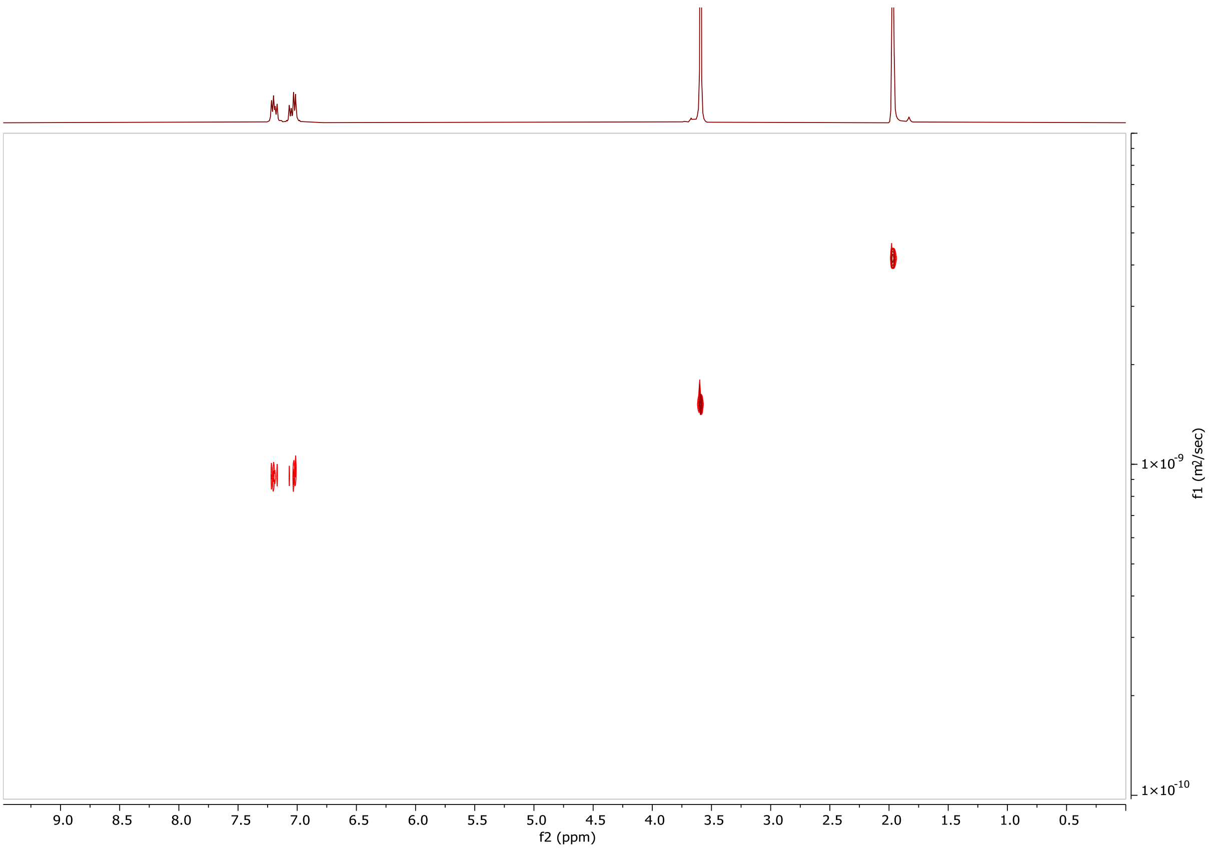

Figure S25: ^1H DOSY NMR (CD_3CN) spectrum of [K(18c6)]$_2$[**6**].

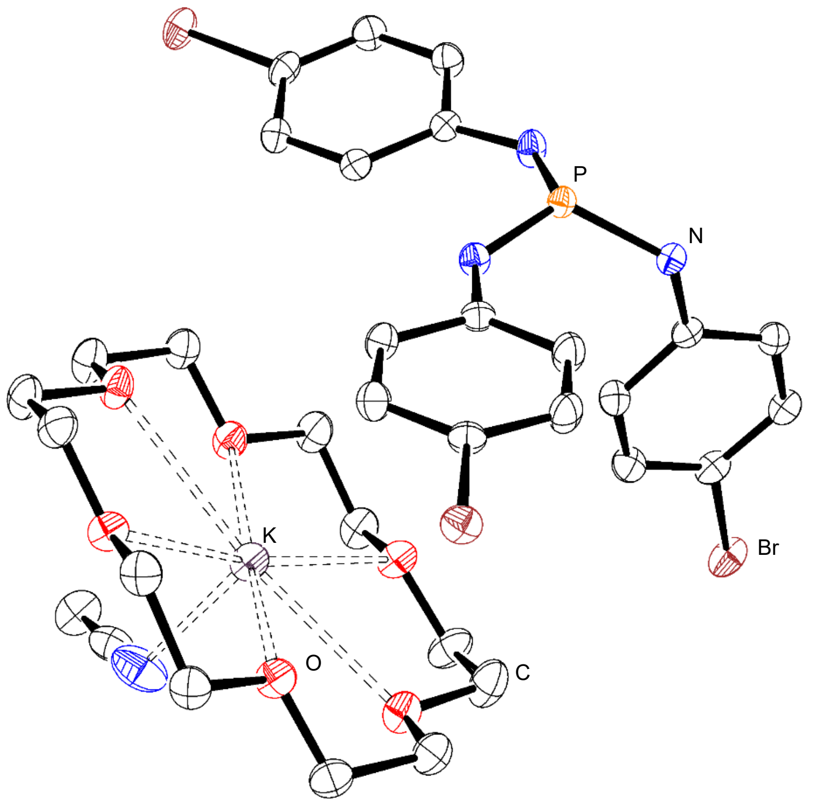

Figure S26: Molecular structure of [K(18c6)]$_2$[**6**] (asymmetric cell). Anisotropic displacement ellipsoids pictured at 50% probability. Anisotropic displacement ellipsoids pictured at 50% probability. Hydrogen atoms omitted for clarity. Nitrogen: blue. Carbon: white. Bromibne: brown. Phosphorus: orange. Oxygen: red. Potassium: violet.

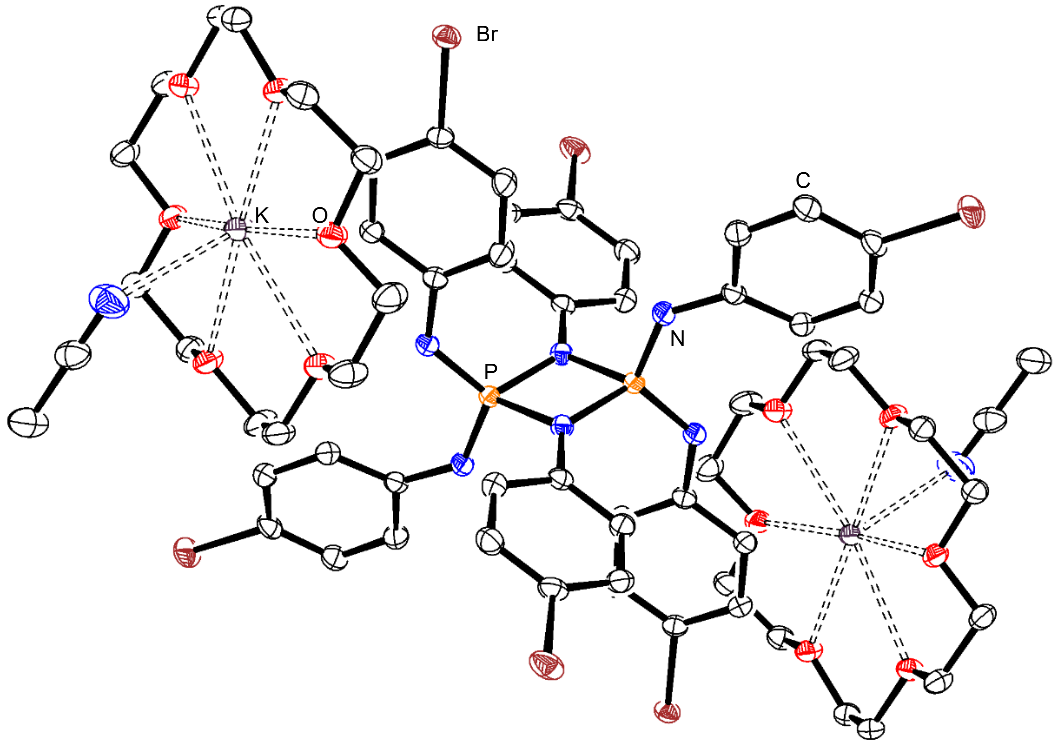

Figure S27: Molecular structure of $[K(18c6)]_2$[**6**] (fragment grown). Anisotropic displacement ellipsoids pictured at 50% probability. Anisotropic displacement ellipsoids pictured at 50% probability. Hydrogen atoms omitted for clarity. Nitrogen: blue. Carbon: white. Bromine: brown. Phosphorus: orange. Oxygen: red. Potassium: violet.

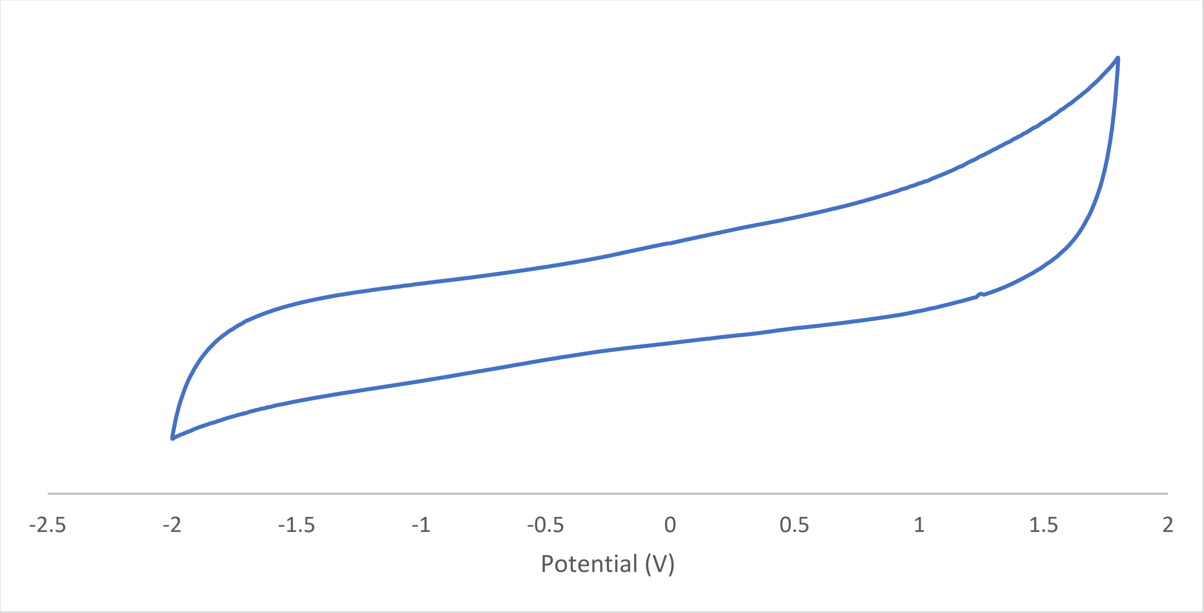

Figure S28: Cyclic voltammetry of $[K(18c6)]_2$[**6**] (3 mM, MeCN, $[Bu_4N][PF_6]$ 100 equiv.). Electrodes: Working – glassy carbon. Counter – platinum wire. Reference – Ag /AgCl (leak proof).

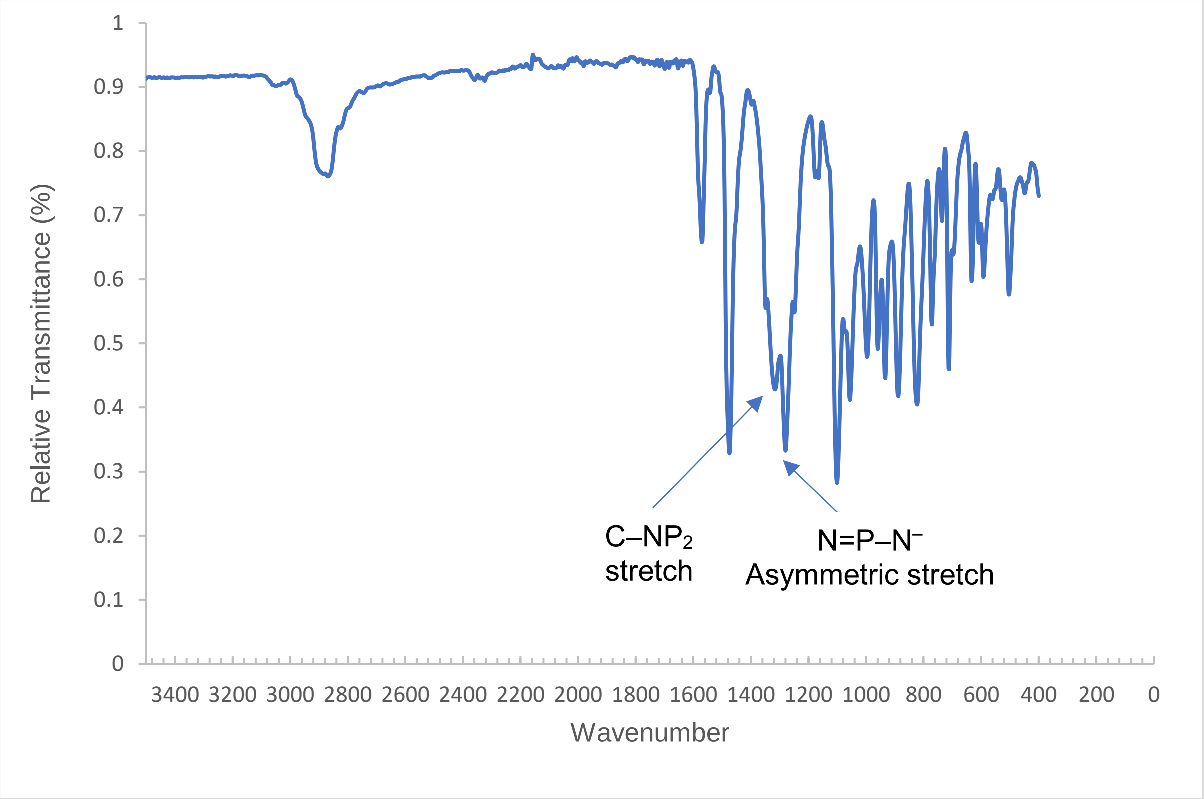

Figure S29: Infrared spectrum of [K(18c6)]$_2$[**6**].

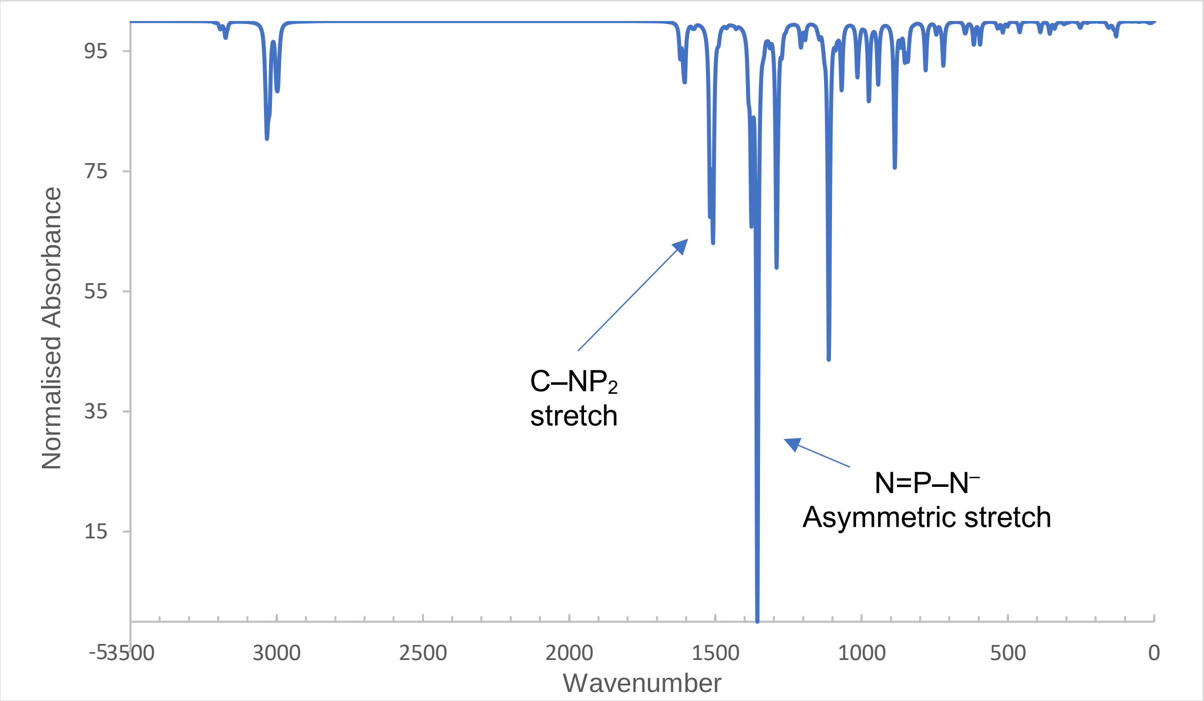

Figure S30: Calculated infrared spectrum of [K(18c6)]$_2$[**6**].

4. Stoichiometric reactivity of $[K(18c6)]_2[\mathbf{1}]$

4.1 Reactivity of [K(18c6)][**1a**] with CO_2

In a J-young NMR tube, [K(18c6)][**1a**] (20 mg, 0.014 mmol) dissolved in DMF-d_7 was degassed by freeze pump thaw cycles. $^{13}CO_2$ (1 atm) was added and the NMR tube was shaken for 1 minute before analyzing by ^1H and $^{13}C\{^1H\}$ NMR spectroscopy. Crystals suitable for SC-XRD analysis formed from the DMF solution overnight and confirmed the formation of [K(18c6)][**3**]. A white insoluble solid also formed, which partially dissolved in CH_3OH and was presumed to be a mix of soluble and insoluble As_xO_y.

NMR Conversion: 100%

^{1}H NMR (DMF-d_7): δ = 7.62 (d, $^3J_{H\text{-}H}$ = 8.8 Hz, 2H, Ar*H*), 7.16 (d, $^3J_{H\text{-}H}$ = 8.8 Hz, 2H, Ar*H*), 3.63 (s, 24H, 18c6) ppm.

^{13}C{^{1}H} NMR (DMF-d_7): δ = 159 (s, $N^{13}CO_2$) ppm.

Mass Spectrometry (DMF): $[C_6{}^{13}CH_5BrNO_2+Na]^-$ Calculated: 237.9440 Found: 237.9495

Mass Spectrometry (CH_3OH): $[AsO_2]^-$ Calculated: 106.9114 Found: 106.9121

$[AsO_2Na]^-$ Calculated: 129.9012 Found: 129.9068

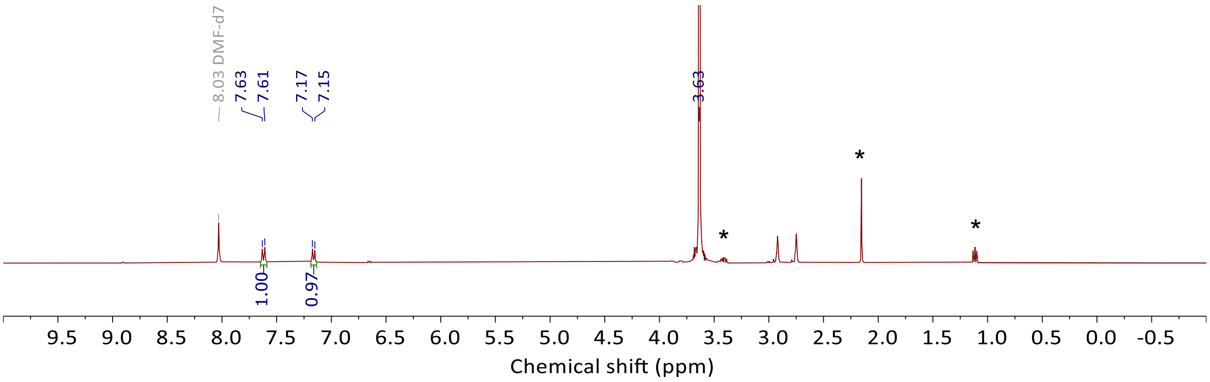

Figure S31: ^{1}H NMR (DMF-d_7) spectrum of [K(18c6)][**1a**] after addition of $^{13}CO_2$ with residual water and THF marked with *.

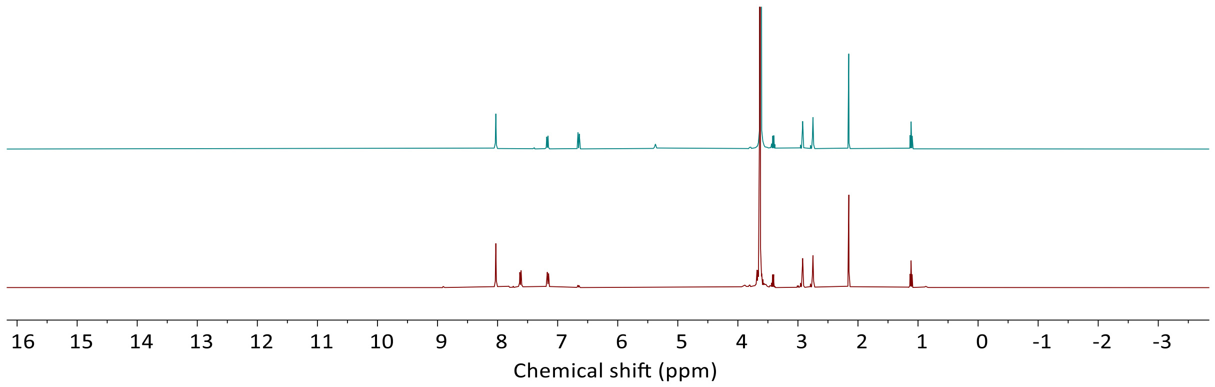

Figure S32: Stacked ^{1}H NMR (DMF-d_7) spectrum of [K(18c6)][**1a**] before addition of $^{13}CO_2$ (top) and after addition (bottom).

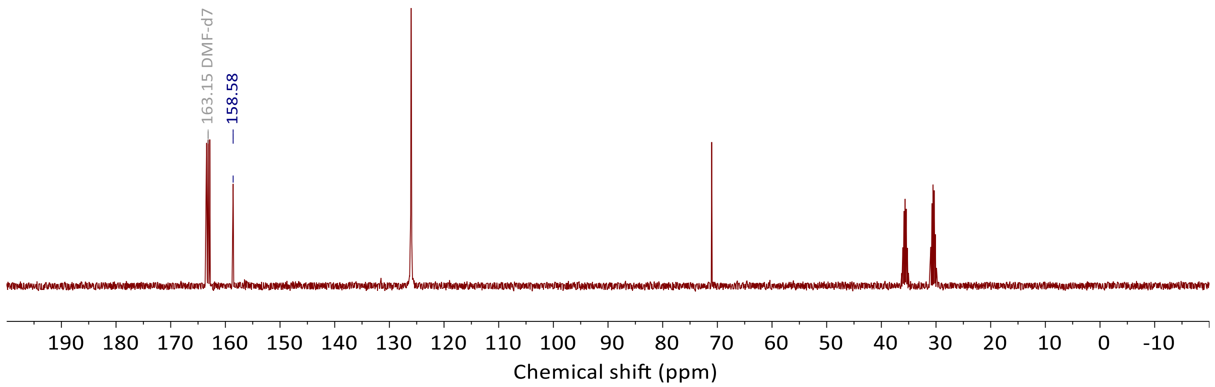

Figure S33: $^{13}C\{^{1}H\}$ NMR (DMF-d_7) spectrum of [K(18c6)][**1a**] after $^{13}CO_2$ addition with the diagnostic resonance corresponding to [K(18c6)][**3**] peak picked.

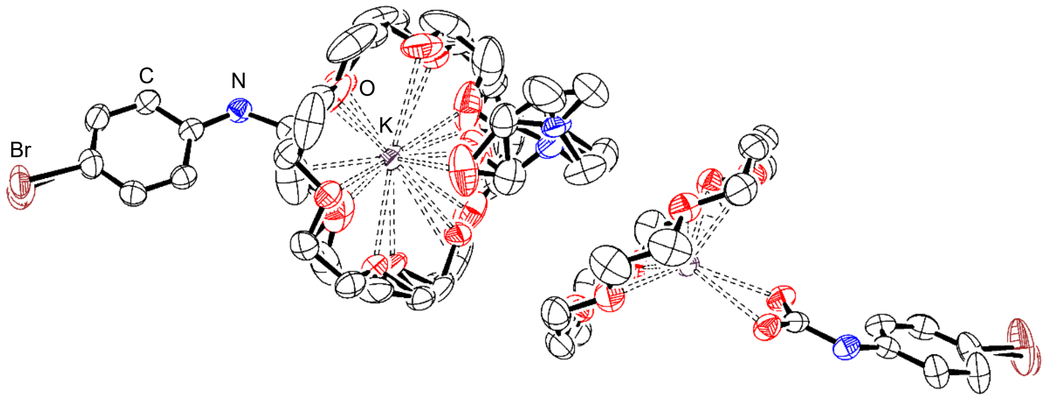

Figure S34: Molecular structure of [K(18c6)][**3**] (asymmetric unit cell). Anisotropic displacement ellipsoids pictured at 50% probability. Hydrogen atoms omitted for clarity. Nitrogen: blue. Carbon: white. Bromine: brown. Oxygen: red. Potassium: violet.

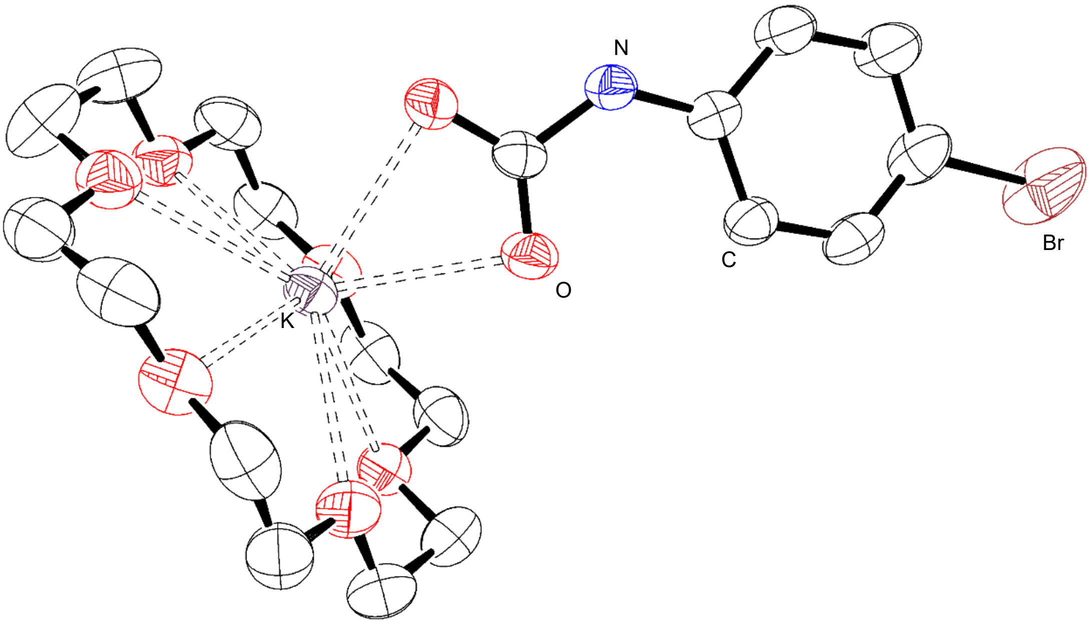

Figure S35: Molecular structure of [K(18c6)][**3**] (single unit). Anisotropic displacement ellipsoids pictured at 50% probability. Hydrogen atoms and DMF omitted for clarity. Nitrogen: blue. Carbon: white. Bromine: brown. Oxygen: red. Potassium: violet.

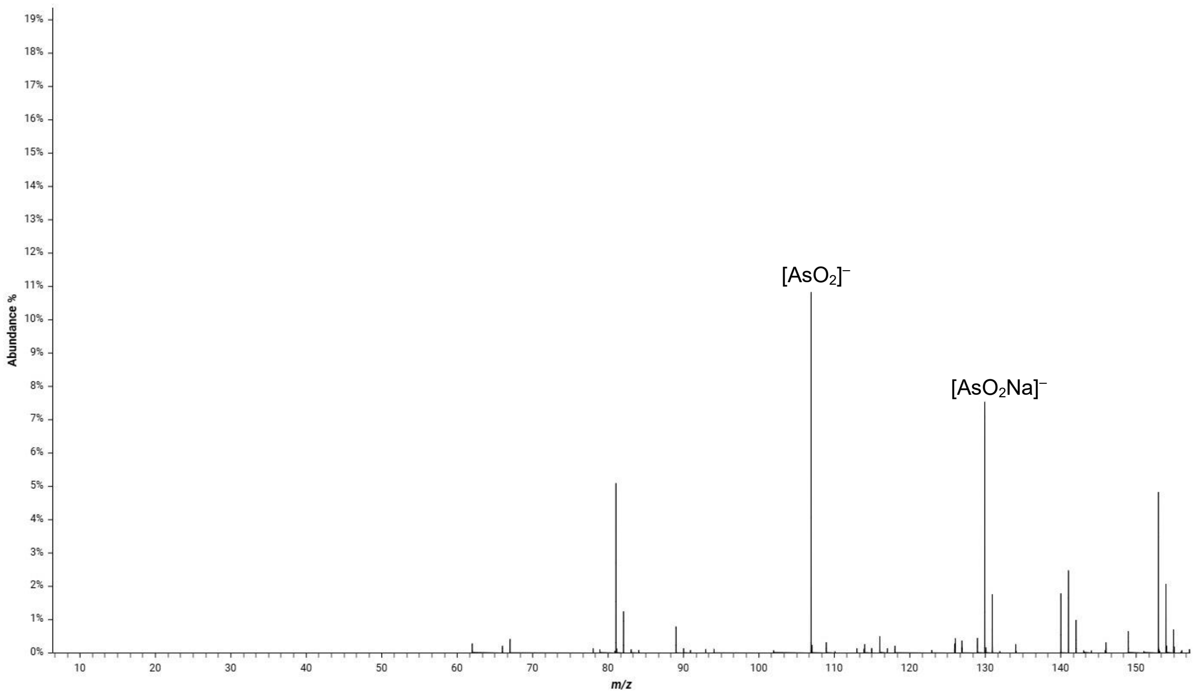

Figure S36: Mass spectrometry (CH_3OH) of the insoluble material from reaction 4.1.

4.2 Reactivity of [K(18c6)][**1a**] with CS_2

In a vial, [K(18c6)][**1a**] (20 mg, 0.014 mmol) dissolved in bench stored CS_2 (0.5 mL) was left to react for 1 hour forming a white solid (presumed to be As_xS_y). The solution was filtered and CS_2 solvent removed, yielding a yellow solid. This solid was dissolved in $CDCl_3$ and analyzed by NMR spectroscopy showing complete conversion to one *p*-BrC_6H_4R containing product that was confirmed to be **4** by SC-XRD on crystals grown from a concentrated CS_2 solution at –18°C. The white solid that initially precipitated was extracted with $CDCl_3$ and analyzed by ^1H NMR and showed only 18c6 with some insoluble material presumed to be As_xS_y. The sequestered salt by-product could not be isolated cleanly from **4**.

^{1}H NMR ($CDCl_3$): δ = 7.50 (d, $^3J_{H\text{-}H}$ = 8.8 Hz, 2H, Ar*H*), 7.36 (d, $^3J_{H\text{-}H}$ = 8.8 Hz, 2H, Ar*H*), 3.56 (s, 24H, 18c6) ppm.

Mass Spectrometry (DMF):

[$C_{13}H_9Br_2N_2S$]$^-$ Calculated: 384.8838 Found: 384.8868.

[AsS_2]$^-$ Calculated: 138.8663 Found: 138.8631

[AsS_3]$^-$ Calculated: 170.8378 Found: 170.8377

[AsS_5]$^-$ Calculated: 234.7825 Found: 234.7867

[AsS_6]$^-$ Calculated: 266.7540 Found: 266.7575

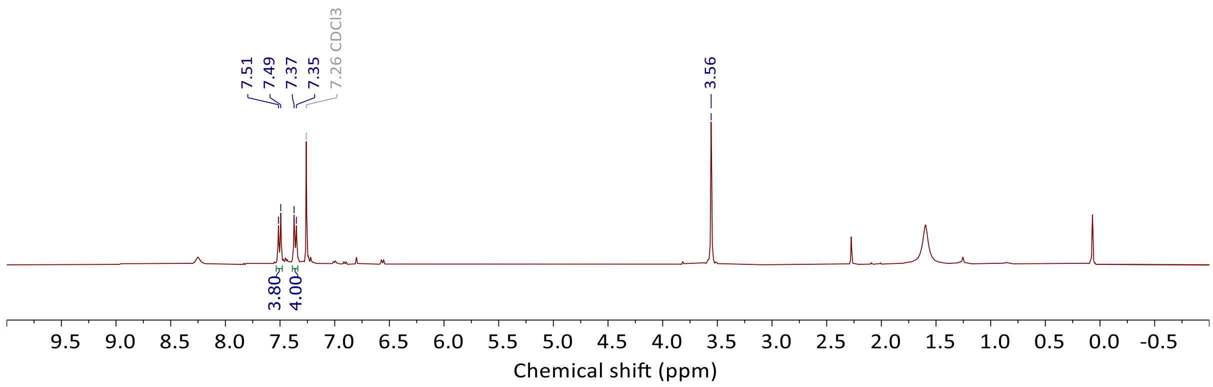

Figure S37: ^1H NMR ($CDCl_3$) of crude soluble material from filtered CS_2.

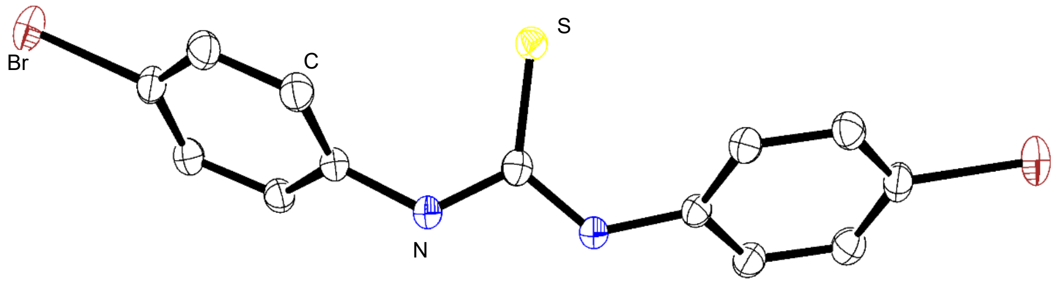

Figure S38: Molecular structure of **4**.[14,15] Anisotropic displacement ellipsoids pictured at 50% probability. Hydrogen atoms omitted for clarity. Nitrogen: blue. Carbon: white. Bromine: brown. Sulfur: yellow.

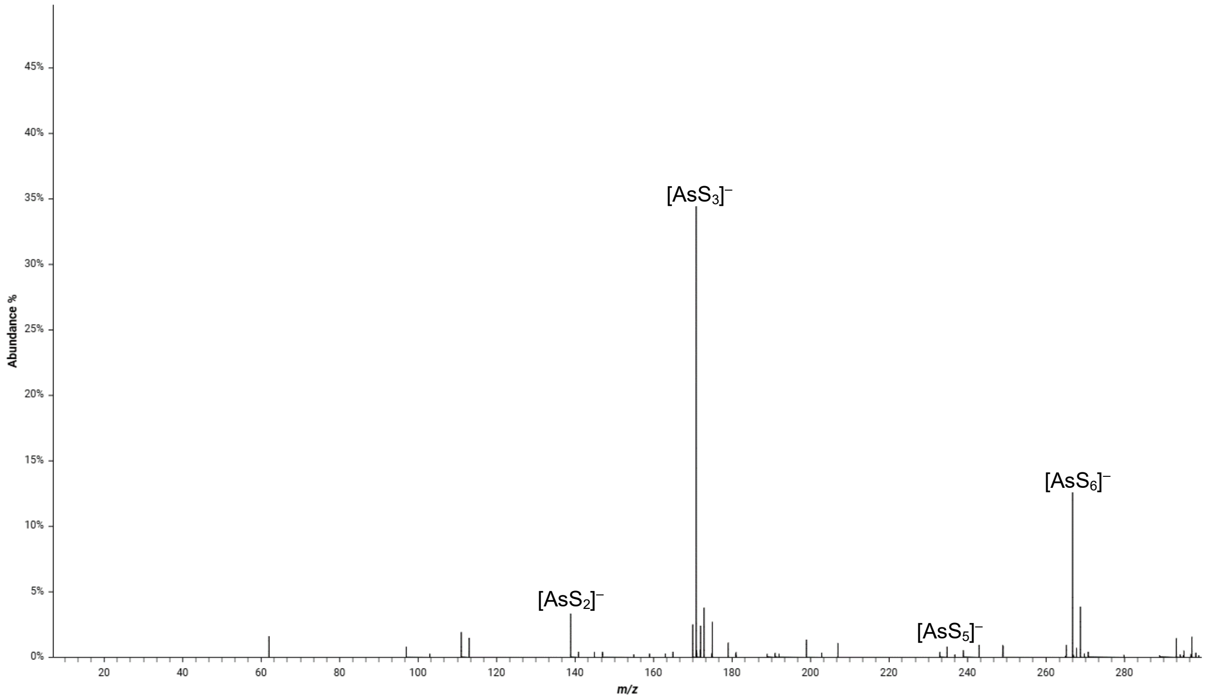

Figure S39: Mass spectrum (DMF) of the insoluble solid formed in reaction 4.2.

4.3 Reactivity of [K(18c6)][**1a**] with 4-IC_6H_4CHO

In a vial, [K(18c6)][**1a**] (20 mg, 0.014 mmol, 1 equiv.) dissolved in MeOD-D_4 was reacted with 4-IC_6H_4CHO (13 mg, 0.056 mmol, 4 equiv.) for 1 hour forming an off-white precipitate which was insoluble in all common laboratory solvents. The solution was filtered and solvent removed before drying at 50°C in vacuo to remove excess 4-IC_6H_4CHO. The remaining solid was analyzed by ^{1}H NMR spectroscopy showing quantitative formation of the corresponding imine (5) with KOH(18c6) detected in solution which could not be cleanly removed.

^{1}H NMR ($CDCl_3$): δ = 8.35 (s, 1H, N*H*), 7.82 (d, 2H, $^3J_{H-H}$ = 8.4 Hz, Ar), 7.60 (d, 2H, $^3J_{H-H}$ = 8.4 Hz, Ar), 7.49 (d, 2H, $^3J_{H-H}$ = 8.7 Hz, Ar), 7.07 (d, 2H, $^3J_{H-H}$ = 8.7 Hz, Ar) ppm.

Mass Spectrometry (MeOH): $[C_{13}H_9BrIN]^+$ Calculated 384.8959 Found: 384.8955

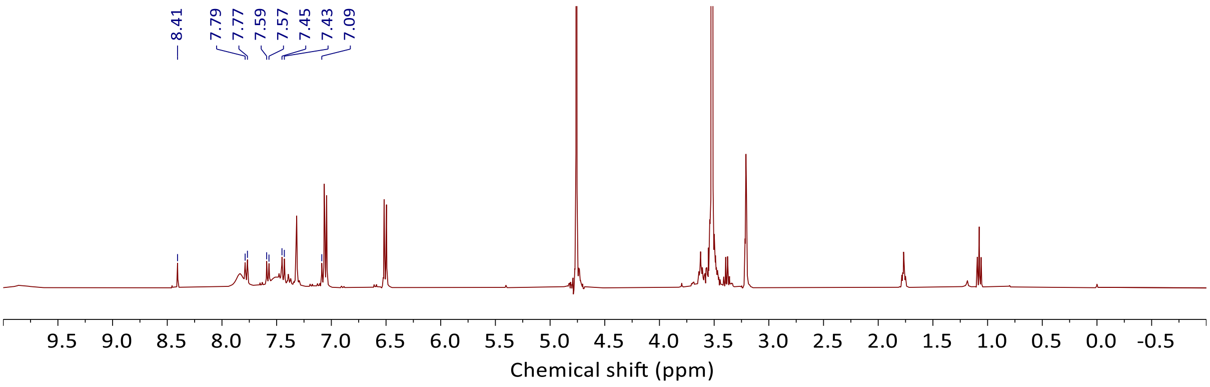

Figure S40: ^{1}H NMR (MeOD-d_4) spectrum of the crude NMR mixture.

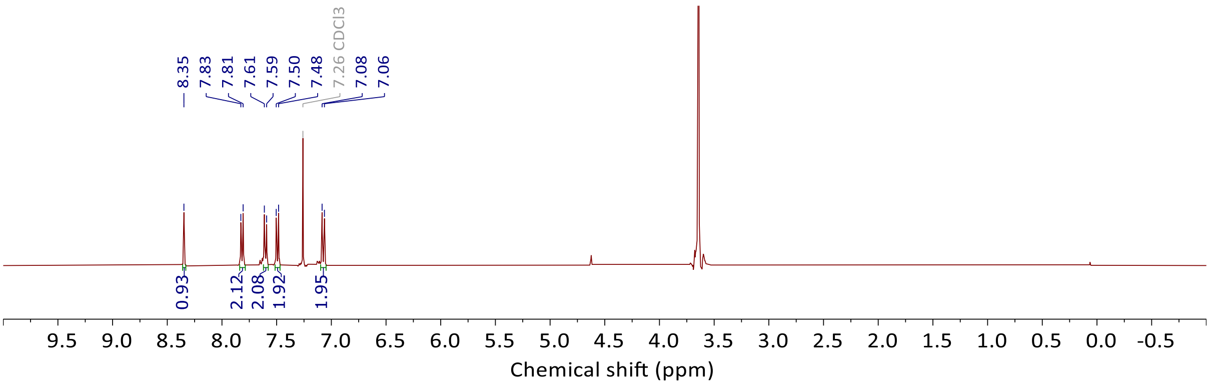

Figure S41: ^{1}H NMR ($CDCl_3$) spectrum of reaction mixture after drying under vacuum at 50°C to remove excess 4-IC_6H_4CHO.[16]

5. Crystallographic Tables

Identification code	[K(18c6)]$_2$[**1b**]	[K(crypt)]$_2$[**1b**]
Empirical formula	$C_{32}H_{48}AsBr_2KN_2O_8$	$C_{30}H_{43}AsBr_2KN_4O_6$
Formula weight	862.56	829.52
Temperature/K	150.15	100.00(10)
Crystal system	monoclinic	monoclinic
Space group	P21/c	P2$_1$/n
a/Å	13.4661(2)	10.66950(10)
b/Å	15.57690(10)	25.6617(3)
c/Å	18.9645(2)	12.94760(10)
α/°	90	90
β/°	90.2920(10)	100.3820(10)
γ/°	90	90
Volume/Å^3	3977.94(8)	3486.98(6)
Z	4	4
ρ_{calc}g/cm^3	1.440	1.580
μ/mm^{-1}	4.824	5.452
F(000)	1760.0	1684.0
Crystal size/mm^3	0.275 × 0.18 × 0.159	0.217 × 0.164 × 0.122
Radiation	Cu Kα (λ = 1.54184)	Cu Kα (λ = 1.54184)
2Θ range for data collection/°	6.564 to 152.802	6.89 to 151.992
Index ranges	-16 ≤ h ≤ 16, -19 ≤ k ≤ 14, -23 ≤ l ≤ 23	-10 ≤ h ≤ 13, -31 ≤ k ≤ 32, -15 ≤ l ≤ 16
Reflections collected	49875	70293
Independent reflections	8275 [Rint = 0.0411, Rsigma = 0.0256]	7210 [R_{int} = 0.0498, R_{sigma} = 0.0297]
Data/restraints/parameters	8275/93/397	7210/0/411
Goodness-of-fit on F^2	1.051	1.036
Final R indexes [I>=2σ (I)]	R_1 = 0.0497, wR_2 = 0.1462	R_1 = 0.0522, wR_2 = 0.1404
Final R indexes [all data]	R_1 = 0.0574, wR_2 = 0.1548	R_1 = 0.0709, wR_2 = 0.1527
Largest diff. peak/hole / e Å^{-3}	1.65/-0.94	1.40/-0.83
CCDC	2515311	2523832

Identification code	[K(crypt)][**2b**]	[K(18c6)][**3**]
Empirical formula	$C_{30}H_{46}AsKN_4O_6$	$C_{41}H_{65}Br_2K_2N_3O_{17}$
Formula weight	672.73	554.99
Temperature/K	100.0(2)	150.00(10)
Crystal system	monoclinic	monoclinic
Space group	$P2_1/n$	$P2_1/n$
a/Å	14.5624(2)	15.1777(2)
b/Å	14.0171(2)	23.9900(3)
c/Å	16.6228(2)	15.5276(2)
α/°	90	90
β/°	105.326(2)	113.305(2)
γ/°	90	90
Volume/Å^3	3272.42(8)	5192.52(14)
Z	4	4
ρ_{calc}g/cm^3	1.365	1.420
μ/mm^{-1}	2.925	3.992
F(000)	1416.0	2304.0
Crystal size/mm^3	0.21 × 0.176 × 0.066	0.26 × 0.23 × 0.16
Radiation	Cu Kα (λ = 1.54184)	Cu Kα (λ = 1.54184)
2Θ range for data collection/°	7.188 to 152.824	6.894 to 152.228
Index ranges	-17 ≤ h ≤ 15, -17 ≤ k ≤ 17, -20 ≤ l ≤ 20	-19 ≤ h ≤ 18, -28 ≤ k ≤ 30, -19 ≤ l ≤ 19
Reflections collected	51331	47694
Independent reflections	6788 [R_{int} = 0.0356, R_{sigma} = 0.0210]	10763 [R_{int} = 0.0375, R_{sigma} = 0.0273]
Data/restraints/parameters	6788/2/389	10763/102/827
Goodness-of-fit on F^2	1.054	1.043
Final R indexes [I>=2σ (I)]	R_1 = 0.0557, wR_2 = 0.1454	R_1 = 0.0386, wR_2 = 0.1004
Final R indexes [all data]	R_1 = 0.0614, wR_2 = 0.1497	R_1 = 0.0508, wR_2 = 0.1103
Largest diff. peak/hole / e Å^{-3}	0.84/-0.65	0.51/-0.42
CCDC	2522284	2515314

Identification code	**4**	[K(18c6)]$_2$[**6**]
Empirical formula	$C_{13}H_{10}Br_2N_2S$	$C_{32}H_{36}Br_3D_3KN_4O_6P$
Formula weight	386.11	888.49
Temperature/K	100.00(10)	100.00(10)
Crystal system	monoclinic	monoclinic
Space group	$P2_1/c$	$P2_1/n$
a/Å	14.09230(10)	12.47880(10)
b/Å	7.08290(10)	16.54380(10)
c/Å	14.0639(2)	17.51130(10)
α/°	90	90
β/°	104.0630(10)	92.4460(10)
γ/°	90	90
Volume/Å^3	1361.71(3)	3611.86(4)
Z	4	4
ρ_{calc}g/cm^3	1.883	1.634
μ/mm^{-1}	8.839	5.991
F(000)	752.0	1784.0
Crystal size/mm^3	0.11 × 0.06 × 0.05	0.256 × 0.122 × 0.069
Radiation	Cu Kα (λ = 1.54184)	Cu Kα (λ = 1.54184)
2Θ range for data collection/°	6.466 to 152.36	7.354 to 151.862
Index ranges	-17 ≤ h ≤ 17, -8 ≤ k ≤ 8, -17 ≤ l ≤ 16	-15 ≤ h ≤ 15, -20 ≤ k ≤ 20, -22 ≤ l ≤ 18
Reflections collected	46344	76951
Independent reflections	2806 [R_{int} = 0.0394, R_{sigma} = 0.0147]	7479 [R_{int} = 0.0394, R_{sigma} = 0.0200]
Data/restraints/parameters	2806/0/163	7479/0/425
Goodness-of-fit on F^2	1.063	1.039
Final R indexes [I>=2σ (I)]	R_1 = 0.0247, wR_2 = 0.0630	R_1 = 0.0244, wR_2 = 0.0591
Final R indexes [all data]	R_1 = 0.0282, wR_2 = 0.0646	R_1 = 0.0271, wR_2 = 0.0603
Largest diff. peak/hole / e Å^{-3}	0.40/-0.52	0.36/-0.61
CCDC	2515313	2515312

6. References

(1) Mamidyala, S. K.; Cooper, M. A. Probing the Reactivity of O-Phthalaldehydic Acid/Methyl Ester: Synthesis of N-Isoindolinones and 3-Arylaminophthalides. *Chem. Commun.* **2013**, *49* (75), 8407–8409. https://doi.org/10.1039/C3CC43838D.

(2) van IJzendoorn, B.; Lister-Roberts, R.; Kaltsoyannis, N.; Mehta, M. Catalytic Nitrous Oxide Degradation with Group 15 Clusters. *J. Am. Chem. Soc.* **2025**, *147* (33), 30317–30325. https://doi.org/10.1021/jacs.5c09618.

(3) CrysAlis PRO. Agilent Technologies Ltd: Yarnton O, England 2014.

(4) Sheldrick, G. M. SHELXT – Integrated Space-Group and Crystal-Structure Determination. *Acta Cryst A* **2015**, *71* (1), 3–8. https://doi.org/10.1107/S2053273314026370.

(5) Dolomanov, O. V.; Bourhis, L. J.; Gildea, R. J.; Howard, J. a. K.; Puschmann, H. OLEX2: A Complete Structure Solution, Refinement and Analysis Program. *J Appl Cryst* **2009**, *42* (2), 339–341. https://doi.org/10.1107/S0021889808042726.

(6) Hohenberg, P.; Kohn, W. Inhomogeneous Electron Gas. *Phys. Rev.* **1964**, *136* (3B), B864–B871. https://doi.org/10.1103/PhysRev.136.B864.

(7) Kohn, W.; Sham, L. J. Self-Consistent Equations Including Exchange and Correlation Effects. *Phys. Rev.* **1965**, *140* (4A), A1133–A1138. https://doi.org/10.1103/PhysRev.140.A1133.

(8) Peng, C.; Ayala, P. Y.; Schlegel, H. B.; Frisch, M. J. Using Redundant Internal Coordinates to Optimize Equilibrium Geometries and Transition States. *Journal of Computational Chemistry* **1996**, *17* (1), 49–56. https://doi.org/10.1002/(SICI)1096-987X(19960115)17:1%253C49::AID-JCC5%253E3.0.CO;2-0.

(9) Gaussian 09, R. A., M. J. Frisch, G. W. Trucks, H. B. Schlegel, G. E. Scuseria, M. A. Robb, J. R. Cheeseman, G. Scalmani, V. Barone, G. A. Petersson, H. Nakatsuji, X. Li, M. Caricato, A. Marenich, J. Bloino, B. G. Janesko, R. Gomperts, B. Mennucci, H. P. Hratchian, J. V. Ortiz, A. F. Izmaylov, J. L. Sonnenberg, D. Williams-Young, F. Ding, F. Lipparini, F. Egidi, J. Goings, B. Peng, A. Petrone, T. Henderson, D. Ranasinghe, V. G. Zakrzewski, J. Gao, N. Rega, G. Zheng, W. Liang, M. Hada, M. Ehara, K. Toyota, R. Fukuda, J. Hasegawa, M. Ishida, T. Nakajima, Y. Honda, O. Kitao, H. Nakai, T. Vreven, K. Throssell, J. A. Montgomery, Jr., J. E. Peralta, F. Ogliaro, M. Bearpark, J. J. Heyd, E. Brothers, K. N. Kudin, V. N. Staroverov, T. Keith, R. Kobayashi, J. Normand, K. Raghavachari, A. Rendell, J. C. Burant, S. S. Iyengar, J. Tomasi, M. Cossi, J. M. Millam, M. Klene, C. Adamo, R. Cammi, J. W. Ochterski, R. L. Martin, K. Morokuma, O. Farkas, J. B. Foresman, and D. J. Fox, Gaussian, Inc., Wallingford CT, 2016.

(10) Perdew, J. P.; Ernzerhof, M.; Burke, K. Rationale for Mixing Exact Exchange with Density Functional Approximations. *J. Chem. Phys.* **1996**, *105* (22), 9982–9985. https://doi.org/10.1063/1.472933.

(11) Perdew, J. P.; Burke, K.; Ernzerhof, M. Generalized Gradient Approximation Made Simple. *Phys. Rev. Lett.* **1996**, *77* (18), 3865–3868. https://doi.org/10.1103/PhysRevLett.77.3865.

(12) Krishnan, R.; Binkley, J. S.; Seeger, R.; Pople, J. A. Self-consistent Molecular Orbital Methods. XX. A Basis Set for Correlated Wave Functions. *J. Chem. Phys.* **1980**, *72* (1), 650–654. https://doi.org/10.1063/1.438955.

(13) Marenich, A. V.; Cramer, C. J.; Truhlar, D. G. Universal Solvation Model Based on Solute Electron Density and on a Continuum Model of the Solvent Defined by the Bulk Dielectric Constant and Atomic Surface Tensions. *J. Phys. Chem. B* **2009**, *113* (18), 6378–6396. https://doi.org/10.1021/jp810292n.

(14) Phaenok, S.; Nguyen, L. A.; Soorukram, D.; Nguyen, T. T. T.; Retailleau, P.; Nguyen, T. B. Sulfur- and Amine- Promoted Multielectron Autoredox Transformation of Nitromethane: Multicomponent Access to Thiourea Derivatives. *Chemistry – A European Journal* **2024**, *30* (7), e202303703. https://doi.org/10.1002/chem.202303703.

(15) Muhammed, N.; Zia-ur-Rehman; Ali, S.; Meetsma, A. 1,3-Bis(4-Bromophenyl)Thiourea. *Acta Crystallographica Section E*, 2007, *63*, o632–o633.

(16) Mehta, M.; Lister-Roberts, R.; Galano, D.; IJzendoorn, B. van; Whitehead, G.; Brookfield, A.; Bowen, A. M.; Kaltsoyannis, N. A Crystalline Nitrogen Chain Radical Anion. Research Square February 27, 2025. https://doi.org/10.21203/rs.3.rs-6055289/v1 (accessed 2026-01-19).
